# Spatial hearing training in virtual reality with simulated asymmetric hearing loss

**DOI:** 10.1038/s41598-024-51892-0

**Published:** 2024-01-30

**Authors:** Chiara Valzolgher, Sara Capra, Kevin Sum, Livio Finos, Francesco Pavani, Lorenzo Picinali

**Affiliations:** 1 Center for Mind/Brain Sciences (CIMeC), University of Trento, Corso Bettini 31, 38068 Rovereto, TN Italy; 2 Audio Experience Design (www.axdesign.co.uk), Imperial College London, London, UK; 3 Department of Statistical Sciences, University of Padova, Padova, Italy; 4 Department of Psychology and Cognitive Sciences (DiPSCo), University of Trento, Rovereto, Italy; 5 Centro Interuniversitario di Ricerca “Cognizione, Linguaggio e Sordità” (CIRCLeS), Rovereto, Italy

**Keywords:** Neuroscience, Auditory system, Learning and memory, Sensorimotor processing, Psychology, Human behaviour, Electrical and electronic engineering

## Abstract

Sound localization is essential to perceive the surrounding world and to interact with objects. This ability can be learned across time, and multisensory and motor cues play a crucial role in the learning process. A recent study demonstrated that when training localization skills, reaching to the sound source to determine its position reduced localization errors faster and to a greater extent as compared to just naming sources’ positions, despite the fact that in both tasks, participants received the same feedback about the correct position of sound sources in case of wrong response. However, it remains to establish which features have made reaching to sound more effective as compared to naming. In the present study, we introduced a further condition in which the hand is the effector providing the response, but without it reaching toward the space occupied by the target source: the pointing condition. We tested three groups of participants (naming, pointing, and reaching groups) each while performing a sound localization task in normal and altered listening situations (i.e. mild-moderate unilateral hearing loss) simulated through auditory virtual reality technology. The experiment comprised four blocks: during the first and the last block, participants were tested in normal listening condition, while during the second and the third in altered listening condition. We measured their performance, their subjective judgments (e.g. effort), and their head-related behavior (through kinematic tracking). First, people’s performance decreased when exposed to asymmetrical mild-moderate hearing impairment, more specifically on the ipsilateral side and for the pointing group. Second, we documented that all groups decreased their localization errors across altered listening blocks, but the extent of this reduction was higher for reaching and pointing as compared to the naming group. Crucially, the reaching group leads to a greater error reduction for the side where the listening alteration was applied. Furthermore, we documented that, across blocks, reaching and pointing groups increased the implementation of head motor behavior during the task (i.e., they increased approaching head movements toward the space of the sound) more than naming. Third, while performance in the unaltered blocks (first and last) was comparable, only the reaching group continued to exhibit a head behavior similar to those developed during the altered blocks (second and third), corroborating the previous observed relationship between the reaching to sounds task and head movements. In conclusion, this study further demonstrated the effectiveness of reaching to sounds as compared to pointing and naming in the learning processes. This effect could be related both to the process of implementing goal-directed motor actions and to the role of reaching actions in fostering the implementation of head-related motor strategies.

## Introduction

Identifying the direction of sounds is crucial for perceiving and interacting with the surrounding world. The human brain extracts spatial information about the surrounding environment by interpreting auditory cues, which result from the interaction of sound waves reaching the ears with the head and pinnae^1,2^. However, defining sound localization as an acoustic task is an oversimplification. In our daily lives, we often have multisensory access to sound sources, allowing us to perceive their positions and interact with them using our bodies. For example, we can physically move closer to or further away from a sound source and manipulate sound sources, such as grabbing a phone and moving it toward our ears. This multisensory experience of the acoustic space and our ability to actively interact with sound sources for localization purposes have prompted researchers to investigate different response methods in sound localization tasks^3^ and explore the role of multisensory information in auditory space adaptation and learning processes. Interestingly, recent studies have utilized multisensory information to develop effective training approaches for improving sound localization abilities in the context of altered hearing experience^4,5^. Some training methods involve providing visual feedback about source positions^6,7^, while others leverage multisensory information and active interaction with sound sources. Several studies have demonstrated the benefits of reaching movements and head movements in enhancing sound localization with modified auditory cues^8–16^ (see also^17–20^).

In the last decade, studying the contributions of multisensory and motor cues in this research field was facilitated by the increased use of new technology based on virtual reality. Before that, sound localization has often been studied by adopting experimental settings comprising several speakers located around participants. More recently, sound localization has increasingly been investigated through a fully-virtual approach. In several studies, researchers have exploited acoustic virtual reality and presented spatialized sound through headphones. This is possible thanks to the use of Head-Related Transfer Functions (HRTF), which characterize the spectro-temporal filtering of a series of source positions around a given head (for more information, see^21^). HRTFs can be measured for a given listener, which is often time-consuming and relatively costly. Non-individual or “generic” HRTFs have also successfully been used in the past to simulate binaural listening (a comprehensive review on this matter can be found in^22^). Headphone-based virtual audio has been successfully used to train sound localization skills^12,23^, as well as to simulate hearing deficits^24^ in more complex and controllable settings if compared to techniques that have been typically adopted in previous works, such as monaural ear-plugs^6,7,14,25^ or ear molds^26^.

In a recent study testing normal hearing people in altered listening condition (i.e., one ear plugged), it has been demonstrated that reaching to sounds reduced localization errors faster and to a greater extent as compared to just naming sources’ positions, despite the fact that, in both tasks, participants received feedback about the correct position of sound sources in case of wrong response^14^. However, exactly which aspects have made reaching to sound more effective as compared to naming remained an open question. The rationale behind the reaching-to-sounds benefit hypothesis is that reaching to sounds requires coordinating different effectors (eyes, head, hand) into a common reference frame^27^. In turn, this may result in a more stable (or salient) spatial coding of sound source location and favor the learning of the association between auditory cues and spatial coordinates. Therefore, following this speculation, the key feature of the reaching-to-sounds benefit may be related to the physical movement of the body (the hand) toward the target space. In order to test this hypothesis, it is necessary to compare reaching-to-sounds action to a condition in which the hand is the effector providing the response, but without it reaching toward the space occupied by the target source. For instance, pointing (i.e. indicating a far target) toward a direction requires implementing a motor action with the hand to select a certain distal location of the stimulus (as the action of reaching requires), but without actually reaching the target position. Precisely, when pointing toward a sound source, the target object is not coded as a function of hand position inside the peripersonal space^28,29^. On the contrary, it is coded only with respect to trunk-centered coordinates without necessarily involving a remap of the multisensory spatial representation^30^.

Moreover, it is important to note that motor strategies involving the head implemented by the participants during sound localization tasks could favor the adaptation to an altered hearing experience. Indeed, it has been observed that the reaching-to-sounds benefits were linked to progressively wider head movements to explore auditory space^14^. The crucial role played by head movements in sound localization has been shown by several works in literature in the last decades^31–34^. The studies of these motor behavioral strategies have been favored by advanced experimental settings permitting the possibilities for active listening with^8,9^ and without involving acoustic virtual reality^35,36^.

A further aspect that may contribute to the reaching-to-sounds benefit concerns the auditory adaptation mechanisms subtending this effect. Different types of interaction with sound sources could lead to either transient or stable adaptations to altered auditory cues. For instance, during the course of the task, participants might learn that sounds reach the impaired ear with lower intensity and perform the task accordingly, by re-weighting the altered binaural cues^37,38^. Alternatively, they could succeed in creating a new complex map of the space by learning new correspondences between the auditory cues available and the external space coordinates^25,39^. Testing participants after they were exposed to altered auditory cues to measure whether or not their performance has remained anchored to previous experience (i.e. after effect) could be a first attempt to investigate the adaptation mechanisms, although still explorative and little adopted for stationary sounds as far as we know.

In the present study, we addressed this hypothesis directly by testing three groups of normal hearing participants each while performing sound localization and we measured their performance and head movements during listening. The task comprised 4 blocks: block 1 and 4 were performed while listening in normal hearing condition and block 2 and 3 in altered listening situations simulating a mild-moderate unilateral hearing loss through auditory virtual reality technology. We instructed participants to either name the label positioned above the speaker (naming group), select the speaker by pointing it with a laser pointer held in their hands (pointing group), or reach the target speaker by moving their hand holding the controller (reaching group). All three groups were provided with audio-visual feedback about sound position only in the case of a wrong response. For block 4, they received no feedback on their responses.

To summarize, the goal of this study is to further explore the contribution of reaching to sound sources when adapting to altered auditory cues. To investigate this research question, we tested whether, and to which extent, sound localization improves across block repetition during simulated asymmetrical hearing loss as a function of instruction (naming, pointing, reaching). More specifically, here follows a list of explorations and hypotheses we planned/formulated at the beginning of our work:

1. As documented in several other studies, we expected a decline in participant performance when exposed to asymmetrical mild-moderate hearing impairment, particularly on the ipsilateral side, and anticipated this decrease to be consistent across all groups.
2. We predicted a reduction in localization errors across altered listening blocks for all groups. However, we hypothesized that this reduction would be more pronounced in the Reaching group as compared to Naming, compatibly with what previously observed^14^.
3. Considering error reduction in the Pointing group, we hypothesized that if hand involvement in the interaction is relevant for adaptation, the improvement in the Pointing and Reaching groups would be similar. Conversely, if hand movement towards a specific spatial position (as required in the reaching task) is crucial for improvement, we hypothesized that the decrease in Pointing would be lower compared to the Reaching group.
4. We explored the impact of instructions (naming, pointing, reaching) in relation to the implementation of head movement strategies. Once again, we hypothesized that this variable could further help to differentiating the contribution of involving the hand versus movement toward the space occupied by the sound.
5. We further investigated the effect of instruction (naming, pointing, reaching) on sound localization learning by testing participants in binaural conditions after having experienced simulated asymmetrical hearing loss. We hypothesize that if reaching towards sound sources (or pointing or naming them) lead to a stable change in the acoustic space processing^37^, we should not observe any bias resulting from compensatory response behaviors with respect to any systematic adjustments introduced by the participants during the altered listening condition (i.e. after-effects) in block 4.

## Material and methods

### Participants

Forty-two participants (age: 22.83 ± 2.49, range = [18–28]) took part in the experiment, including 22 males and 20 females. There were 5 left-handed attendees (2 in the pointing group and 3 in the naming group). All methods were performed in accordance with the Declaration of Helsinki (1964, amended in 2013) and participants provided informed consent. The experimental protocol was also accepted by the ethical committee of the University of Trento (protocol: 2022–009). Before proceeding with the experimental task, participants stated that they had no visual or motor deficits. In addition, to exclude hearing deficits, the hearing threshold was recorded by an audiometer (Grason Stadler GSI 17 Audiometer) and different frequencies (250, 500, 1000, 2000, 4000 Hz) were tested separately for both ears. The average threshold among participants was 3.01 ± 3.88 dB HL.

### Apparatus and stimuli

The tools used were a Head-Mounted Display (HMD, Oculus Quest 2), its controller and headphones (over ear headphones Sennheiser HD 650 S, HiFi, frequency range: 10–41.000 Hz). The playback level was calibrated in order to deliver a signal at approximately 60 dB SPL A-weighted measured at the listener’s ears. The experiment took place in a soundproofed and partially anechoic booth (Amplifon G2 × 2.5; floor area = 200 × 250 cm, height = 220 cm; background noise level of the booth during the task: 25–30 dB SPL A-weighted).

The virtual scenario was a square room, developed with platform Unity3D (Unity Technologies, San Francisco, CA), similar in size to the real one. The room was empty, with a door behind participants and designed with exposed bricks. Two lines drawn along the middle of each wall, including the floor and ceiling helped participants to remain in the center of the room during the experiment. Participants saw 17 speakers distributed in a semicircle in front of them at the ears level, at 55 cm from participants’ head and spaced about ± 80° of visual angle (each speaker was positioned 10° apart from the other). Above each speaker, a numerical label from 1 to 17, was located. These labels changed randomly on trial-by-trial basis, to avoid anchoring of responses to the previously seen label, to favor orienting of attention to the location of the new label and to avoid stereotypical responses.

Sound spatialization was performed using the Unity integration of a convolution-based algorithm named 3D Tune-In Toolkit^40^. Sounds were spatialized using the HRTF of a KEMAR dummy head mannequin from the SADIE database^41^, and Interaural Time Differences (ITDs) were customized for each individual user according to their head circumference. In order to simulate nearfield sound sources (i.e. closer than the distance at which the HRTF was measured), the Interaural Level Differences (ILDs) were corrected using a spherical head model, accounting also for the acoustic parallax effect. The acoustic environment (a small room) was simulated using the Reverberant Virtual Loudspeakers (RVL) technique, as implemented in the 3D Tune-In Toolkit and recently assessed in a perceptual evaluation study^42^. The auditory stimulus consisted of a white noise, modulated in amplitude (2 Hz), which was transmitted using headphones and spatialized as it was emitted by one of the 17 loudspeakers. Note that head movements were permitted during the sound emission and that the acoustic stimulus changed coherently with participants’ head movements (i.e. turning their heads or approaching speakers). During the task, two different listening conditions were implemented: binaural or altered. The latter consisted of the simulation of mild-to-moderate unilateral hearing loss, which was obtained again using the 3D Tune-In Toolkit. Signals were processed using a gamma tone-based multiband compressor, emulating a hearing loss of 30 dB HL at 250 Hz, decreasing to 50 dB HL above 1 kHz.

The experiment was controlled by a custom patch built using MaxMSP 8 software (www.cycling74.com). In the Naming condition, the experimenter entered manually the label pronounced by participants corresponding to the speaker from which they thought the sound was emitted whereas in the Reaching and Naming conditions this information was automatically recorded by the software.

### Procedure

**Figure 1:**
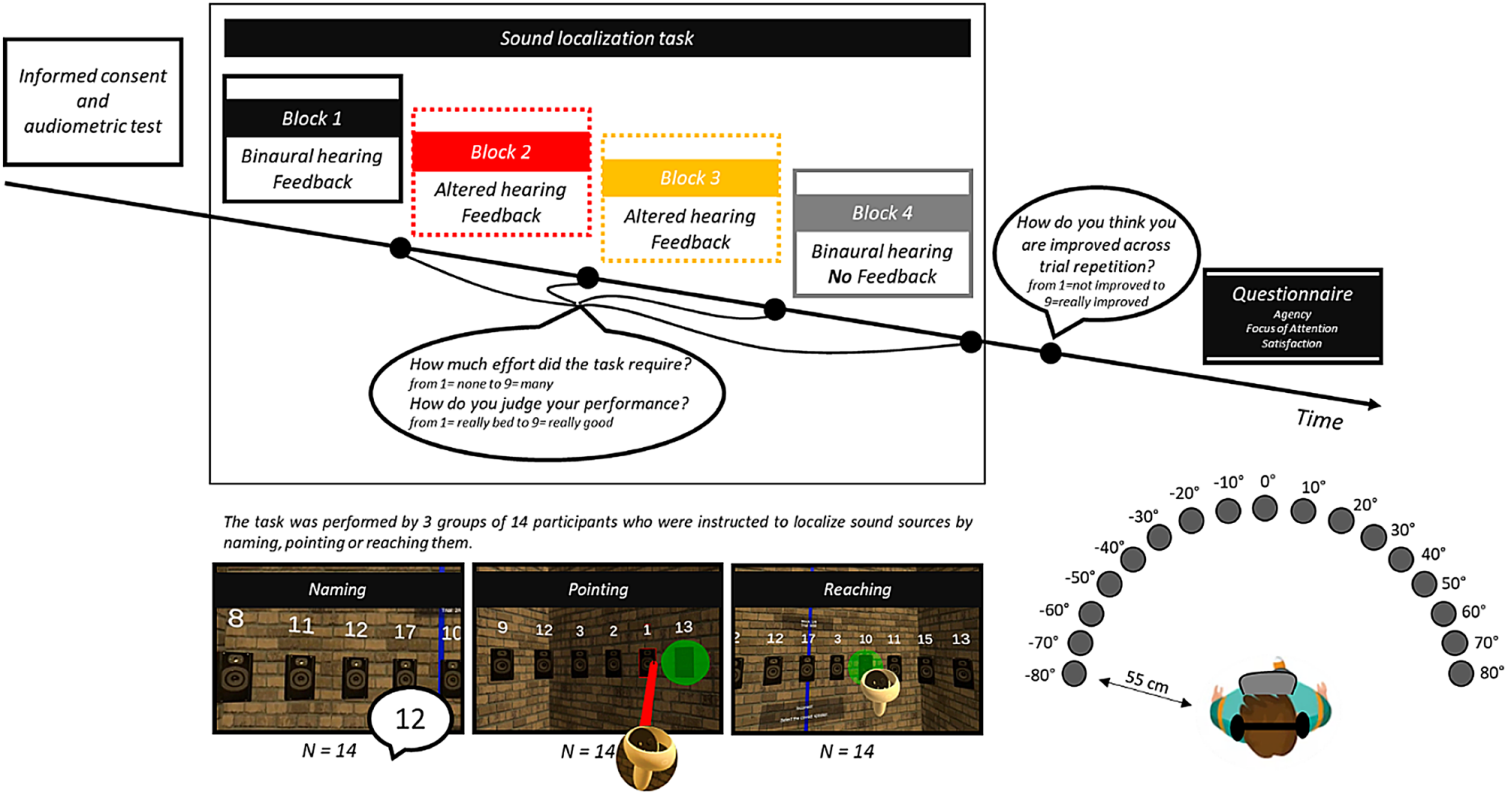
Experimental Timeline and Procedure. Schematic description of the experimental timeline. The first step was signing the consent and taking part in the audiometric examination. Then they performed a sound localization task. The task was composed of 4 consecutive blocks performed in different hearing conditions. Participants were divided in 3 groups, which were instructed to either naming, pointing or reaching the sound sources to localize them. Finally, participants were invited to answer some questions. We insert in the figure, three captures of the visual scene observable by the participants while doing the corresponding task and (to the right) an eye bird view representation of the speakers’ position relative to the head at the beginning of each trial.

Participants, after signing the consent and taking part in the audiometric examination, performed a sound localization task. They were invited to sit on a chair placed in the center of the room, the circumference of their head at the ears plane was measured using a seamstress meter and inserted in the app’s interface, in order to customize the ITDs. Then, they were instructed to wear the head-mounted display and hold the controller in their right hand (note that even the left-handed participants (N = 5) were instructed to use their right hand). All participants were instructed to perform a single sounds localization task following different instructions as a function of their group. Specifically, the sample was divided into 3 groups of 14 people (Fig. 1). The Naming group was instructed to localize sound sources by naming the numerical label located over the speakers. The Pointing group was instructed to localize sound by pointing toward the source by directing toward it a laser pointer emanating from the controller (see Supplementary Video 1). Finally, the Reaching group was instructed to reach the speaker using the controller to localize it (i.e. participants extend their arm and hand holding the controller to move it to the perceived source) (see Supplementary Video 2).

All participants, irrespectively of their group, were informed that they were free to move in the space as they want during the sound emission as in our previous work ^14,17^. Furthermore, they were apprised that there was no time limitation to deliver the answer and that they could ask for a break whenever they wished. Note that the fact that there was no time limitation permitted participants to listen to the sound as long as they wanted. The mean of the response time (calculated from the beginning of the sound to the first response) was 7.56 s for the Reaching group, 7.49 s for the Pointing group, and 9.83 s for the Naming group. Response time for the naming group was longer because it also comprised the time that the experimenter needed to enter the number in the software. For this reason, we did not use this value as a dependent variable. The experimental design includes a total of 4 blocks of 68 trials (4 trials for each source) which were preceded by 2–3 test trials when needed to explain the instructions: the first and the fourth were performed in binaural listening condition, while the second and the third in altered listening condition (i.e. a single ear mild moderate hearing loss). Eight participants for each group had a loss in the right ear, while 6 participants for each group in the left one (see^43,44^ and note that Rabini et al., did not find any differences in alteration effect by plugging the left or the right ear). For each trial, the starting position consisted in orienting the head toward the central speaker (marked with a cross) and remaining still with the head until the beginning of the sound. The system delivered the sound only when the head was oriented toward the center of the speaker array. In the first three blocks, once located the speaker, if the answer was correct the sound stopped. Otherwise, the sound continued, and a green flash was provided to highlight the correct speaker. At this time, participants had to select the correct speaker to stop the sound and finish the trial. Conversely, in the last block (performed in binaural listening) no feedback was provided to record possible after-effects. Note that whenever a response was provided (irrespective of the task instruction and response correctness) the controller in the participant's hand vibrated.

At the end of each block, we asked participants to have a break and remove their helmets. During the pause, we asked them to evaluate the amount of effort required during the task (*“How much effort did the task require from 1* = *none to 9* = *many?”*) and their judgments about their performance *(“How do you judge your performance from 1* = *really bad to 9* = *really good?”*) on Likert scales from 1 to 9. At the end of the experiment, we interview them verbally about the strategies they have adopted to solve the task and their performance improvement from 1 to 9 during the altered hearing condition (block two and three) *(“How do you think you are improved across trial repetition? from 1* = *not improved to 9* = *really improved?”*).

Afterward, participants carried out a questionnaire which served to investigate their subjective experience in virtual reality and the agency on the sound they felt during the task, irrespectively of listening conditions as well as their focus of attention and satisfaction in doing the task (15 items adapted from the work of Wiebe^45^. Participants were asked to report their agreement evaluation (from 1, absolutely disagree, to 9, absolutely agree) to a series of items regarding agency, attention, and satisfaction (results are reported in the Supplementary Information 2).

### Analysis

To document the performance, we measured the absolute and signed errors along the horizontal dimension obtained by calculating the discrepancy between the position of the speaker and participants’ responses for each trial respectively in absolute or signed values. The signed error was calculated by subtracting the position along the horizontal dimension of the speaker from the position of the response (i.e. response – speaker): negative values mean that participants perceived the sound as shifted toward left and positive values toward right. To further describe participants performance, we also measured the % of correctness of participant responses and the results are available in the SM.

To describe head movements, we considered head rotation around the vertical axis as they are the ones more relevant for our task as they can change binaural cues at the two ears and measured the number of reversals during the trial (i.e. the changes in the angular direction of head rotation around the vertical axis) and the head-rotation extent around the vertical axis. We counted as a movement all reversals wider than 5 degrees to exclude movements that are not indicators of spontaneous head intentional movements and may reflect micro postural movements not related to the task. To calculate head-rotation extent we sum the absolute value of the rightward and leftward head rotation extremity. Furthermore, we also calculated a measure of approaching behavior. These indices represent the values in centimeters of the minimum 2d distance between the head position and the speaker emitting the sounds: zeros represented the starting distance at the beginning of the trial (about 55 cm), negative values represented the reduction of distance in centimeters (i.e. approaching). Note that when considering the independent variable *speaker position* we referred to the positions in degrees in azimuth having as reference the initial position of the head (Fig. 1). As the here proposed sound localization task was dynamic and head movements were permitted, these relative positions changed during the trial as a function of participants’ head movements.

Generalized Linear mixed-effect (GLME) and linear mixed-effect (LME) modeling and nonparametric tests were used for statistical analyses. Statistical analyses were run by using R (version 1.0.143). For the GLME model, we used the R-packages emmeans, lme4, lmerTest in R Studio^46,47^. The R-package car was used to obtain deviance tables from the LME models, which permitted the description of the data in terms of main effect and interaction by reporting the Chi-Square value and the respective p-values. We investigated interactions by means of contrasts functions in the emmeans package. As standard in statistical literature, inference in LME model relies on t distribution with approximated degrees of freedom, while z distribution is used for other GLME models. Note that we adopted the Poisson distribution when the dependent variables were not normally distributed, and they involved counted data (e.g. number of reversals). We also use it to interpret absolute errors which can be investigated statistically by considering them as the number of places by which the given answer deviated from the correct one (e.g. “he was wrong by 2 places”). Note that during our analysis of the space head-rotation extent and approaching behavior, when necessary, we corrected the skewness of distributions by log-transforming the variables. Furthermore, when running the analysis on head variables, we did not consider the trials in which the sound was emitted by the central speaker of the array (about 6%, in Fig. 4 we reported the means by plotting the point, but we did not comprise it when plotting the smooth lines). Furthermore, when running the analysis considering the approaching index as depend variable, in order to fit the correct distribution (i.e. we have log-transformed the depend variable) we did not include the values in which participants did not move (i.e. index is zero), which was about 4% of trials. Codes and data can be retrieved from osf.io/4pykg.

## Results

### Sound localization during virtual binaural hearing as a function of instruction (naming, pointing, reaching)

**Figure 2:**
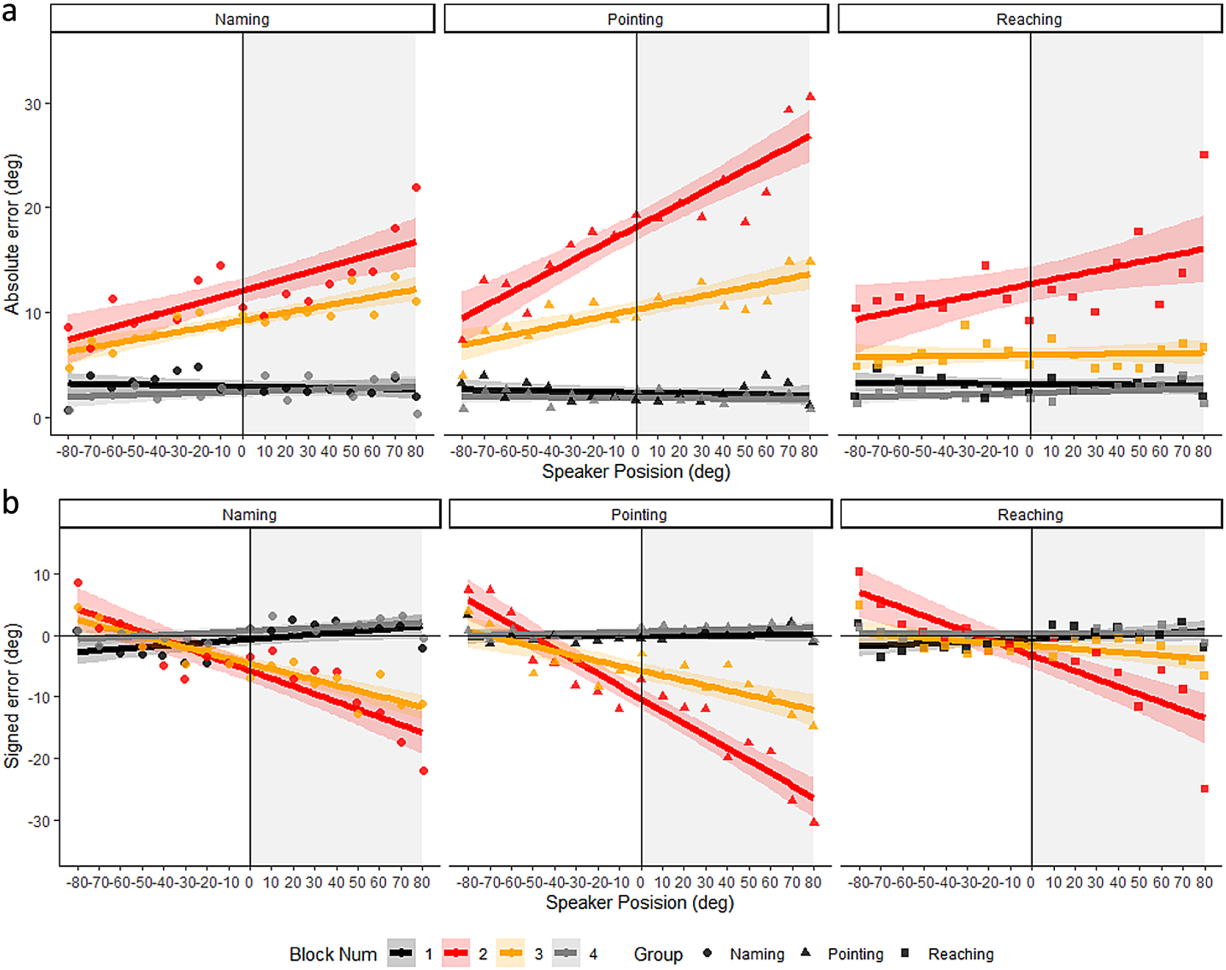
Participants' performance. Absolute error (**A**) and signed error (**B**) across the four blocks (1: black; 2: red; 3: orange and 4: grey) and as a function of speaker position (x axis) and group (Naming: to the left, points; Pointing: center of the figure, triangles; Reaching: to the right, squares). Linear regression (solid line), with 95% confidence intervals.

To investigate if sound localization during virtual binaural hearing changed as a function of instruction, we focused on the first block of the experiment (in black in Figs. 2 and 4).

#### Performance errors

We entered absolute error in a GLME model with speaker position (considered as a continuous variable) and group (naming, pointing or reaching) as fixed effects. To account for the variability related to individual participants, we also included participants (intercept and slope) as random effects. No effects were observed (all *ps* > 0.47) (Fig. 2A, note that we considered in the analysis the difference in terms of number of speaker positions between the response and the correct speaker which was calculated as absolute error/10° (count), while in the Figures, we directly reported absolute error). Instead, analysis on signed error revealed a main effect of speaker position (*X*^2^ (1) = 8.54, *p* = 0.003), suggesting that participants reported the sound as slightly shifted toward the center of the speaker array (Fig. 2B). However, this occurred irrespective of Group, hence the localization instructions. We can tentatively argue that the three groups demonstrated similar abilities to localize sounds under unaltered auditory conditions.

#### Subjective judgments

**Figure 3:**
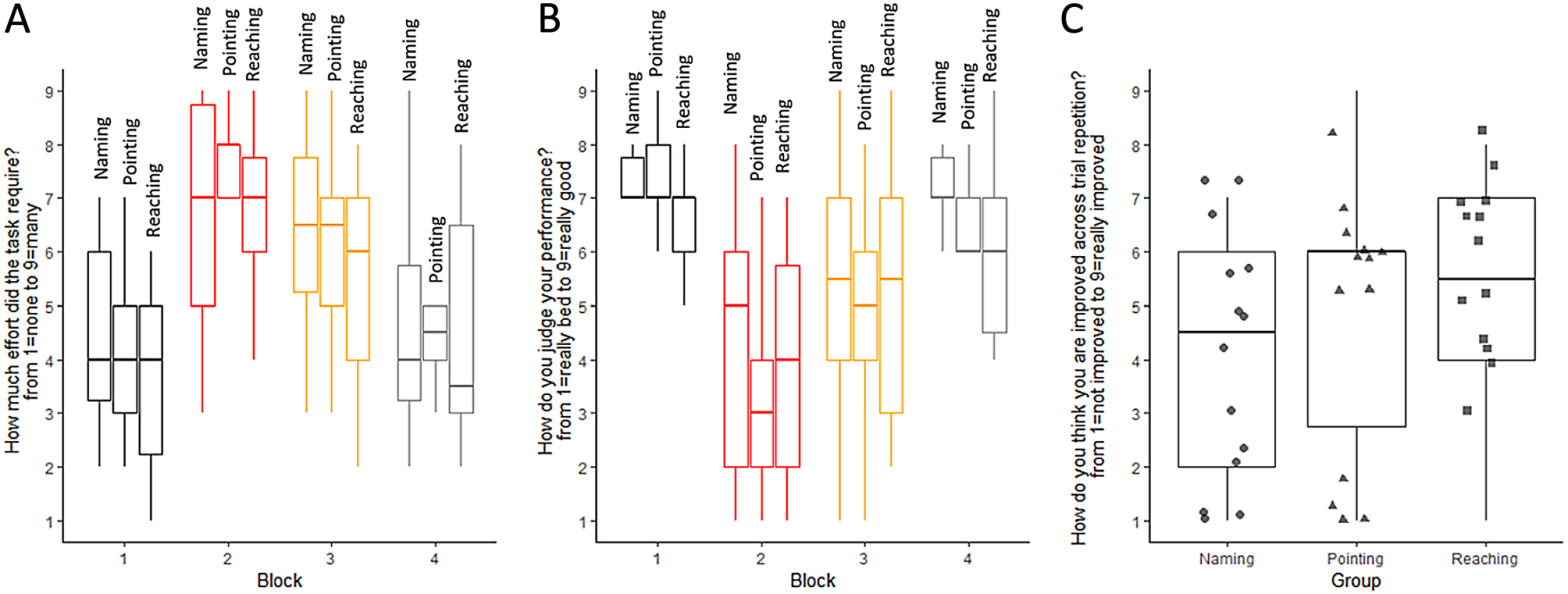
Participants' subjective judgments. Perceived effort (**A**), judgment about performance (**B**) and improvement (**C**) across the four blocks (1: black; 2: red; 3: orange and 4: grey) and as a function group (Naming: to the left, points; Pointing: center of the figure, triangles; Reaching: to the right, squares). Standard errors were reported.

We did not observe any effects of group on perceived effort (Fig. 3A, Kruskal–Wallis Test: *H*(2) = 0.98, *p* = 0.61) nor for the other subjective judgements about performance (Fig. 3B, Kruskal–Wallis Test: *H*(2) = 2.69, *p* = 0.26).

#### Head-related behavior

**Figure 4:**
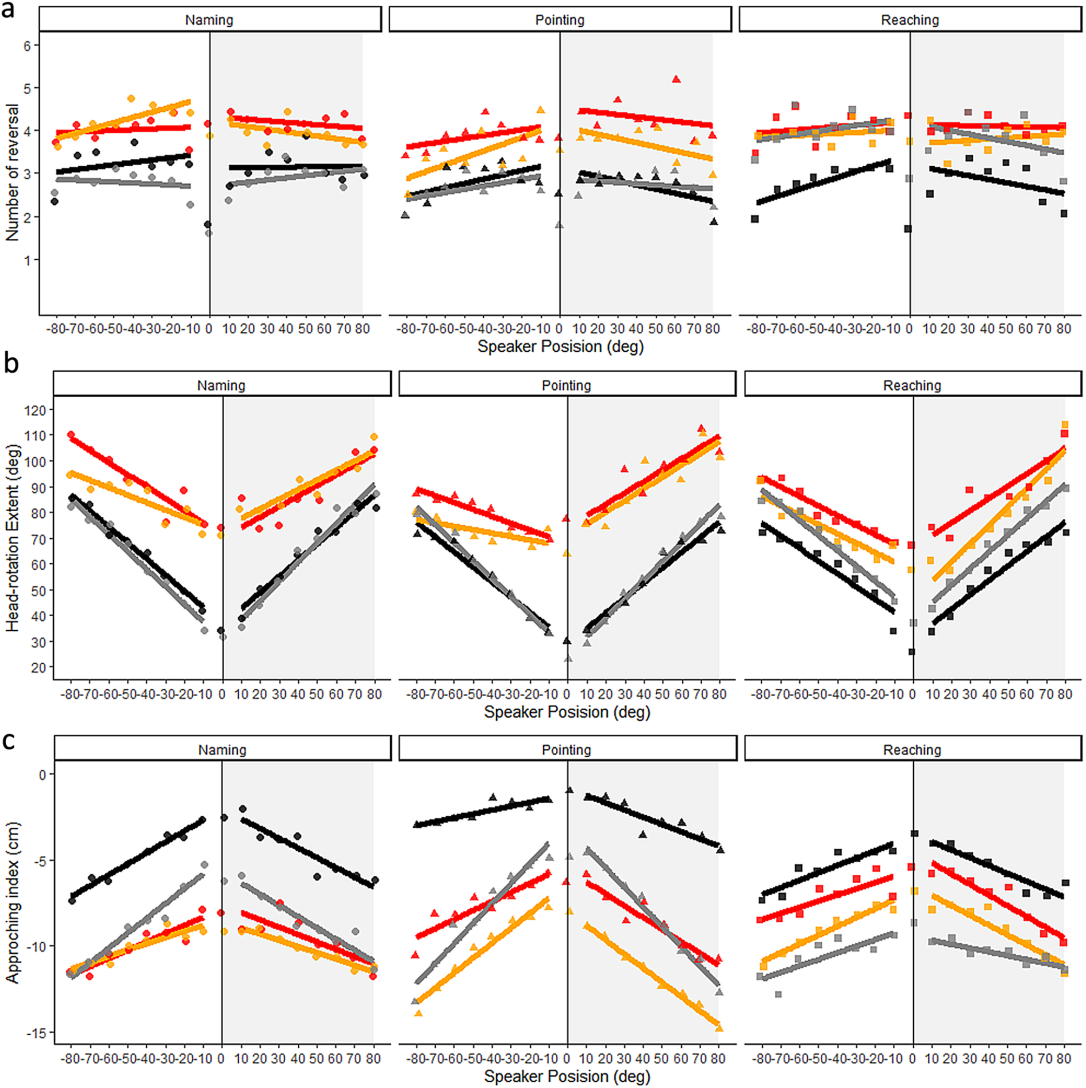
Head-related behavior. Number of reversal (**A**), head-rotation extent (**B**) and approaching index (**C**) across the four blocks (1: black; 2: red; 3: orange and 4: grey) and as a function of speaker position (x axis) and group (Naming: to the left, points; Pointing: center of the figure, triangles; Reaching: to the right, squares). Linear regression (solid line). We did not include confidence intervals to increase the legibility of the graph.

We entered number of reversals in a GLME model (family Poisson) with speaker position, side (left, right) as fixed effects and participant (slope and intercept) as random effects. We did not observe any effects (all *ps* > 0.28) (Fig. 4A). A similar LME analysis on head-rotation extent revealed a main effect of speaker position (*X*^2^ (1) = 71.74, *p* < 0.001) suggesting that space explored by the head increased when sounds were emitted by peripheral speakers (Fig. 4B). Considering the approaching index, we found a main effect of speaker position (*X*^2^ (1) = 30.00, *p* < 0.001) and interaction between side and group (*X*^2^ (2) = 15.86, *p* < 0.001). Reaching and naming group approached the speaker more than pointing and the approaching behavior was greater for right speakers as compared to left ones for the pointing group (*t* = 4.67, *p* < 0.001) and for the reaching group (*t* = 2.09, *p* = 0.03), but not for naming (*t* = 0.94, *p* = 0.35) (Fig. 4C). This could possibly be due to the fact that participants always respond using the right hand, but given the very small size of this difference (0.5 cm) we do not discuss this finding further.

These results revealed that participants' performance did not change. However, participants of the Pointing group did not approach the target speaker as the observed other two groups. It is likely that, even during the sound emission, Pointing group participants assumed a posture functional for implementing pointing response, pressing their backs to the chair to make the whole speaker array visible to be able to direct the laser.

### Sound localization when altering hearing experience by simulating asymmetrical hearing loss as a function of instruction (naming, pointing, reaching)

To investigate if sound localization changed when altering hearing experience by simulating asymmetrical hearing loss as a function of instruction, we focused on the difference between the first and the second blocks of the experiment (colored in black and red in Figs. 2 and 4). Note that participants’ response times increased in block 2 (10.6 s) as compared to block 1 (7.17 s) revealing an increase in the task difficulty (*t*(41) = 7.86, *p* < 0.001).

#### Performance errors

We entered absolute error in a GLME model (family Poisson) with listening condition (binaural-block 1 and altered listening-block 2), speaker position (considered as a continuous variable and encoded as if all participants listened with their right ear in an altered manner) and group as fixed effect, and participants (slope and intercepts) as random effects. We documented a main effect of listening condition (*X*^2^ (1) = 429.23, *p* < 0.001) and two interactions between speaker position and listening condition (*X*^2^ (1) = 19.17, *p* = 0.01) and listening condition and group (*X*^2^ (2) = 53.26, *p* < 0.001) (Fig. 2A). Absolute error increased during the altered hearing as compared to the binaural hearing and particularly for the speakers positioned in the side ipsilateral to the ear with the altered hearing experience. Performance decrement was more pronounced for the pointing group (*z* = 28.08, *p* < 0.001) as compared to the naming (*z* = 20.71, *p* < 0.001) and the reaching group (*z* = 21.41, *p* < 0.001). As visible in Fig. 2B, signed error increased during the altered hearing as compared to the binaural hearing (*X*^2^ (1) = 42.32, *p* < 0.001) and particularly for the speakers positioned in the side ipsilateral to the ear with the altered hearing experience (*X*^2^ (1) = 88.96, *p* = 0.01). Again, performance decrement was more pronounced for the pointing group (*t* = 12.97, *p* < 0.001) as compared to the naming (*t* = 6.51, *p* < 0.001) and the reaching group (*t* = 3.41, *p* < 0.001; *X*^2^ (2) = 47.61, *p* < 0.001). Furthermore, we also documented that for the pointing group signed errors increased as a function of speaker position with a greater extent (*t* = 12.63, *p* < 0.001) as compared to naming (*t* = 9.43, *p* < 0.001) and reaching group (*t* = 8.89, *p* < 0.001; a three-way interaction between listening condition, speaker position and group: *X*^2^ (2) = 8.19, *p* = 0.02).

#### Subjective judgments

Considering participants’ rates of perceived effort and judgments about their performance, we did not document any effect of group (all *ps* > 0.26), but we observed an increased in the perceived effort reported during the block 2 (Friedman Test: *X*^2^ (1) = 19.29, *p* < 0.001) and participants reported to performed worse during the block 2 as compared to the first one (Friedman Test: *X*^2^ (1) = 19.67, *p* < 0.001) (Fig. 3).

#### Head-related behavior

We entered number of reversals in a GLME model (family Poisson) with speaker position, listening condition, side as fixed effects and participant (slope and intercept) as random effects. We found a main effect of listening condition (*X*^2^ (1) = 98.89, *p* < 0.001) and a two-way interaction between listening condition and group (*X*^2^ (2) = 17.19, *p* < 0.001). These results suggested that participants increased the number of movements during the altered hearing as compared to the binaural condition. This increment was higher for the reaching (*z* = 14.43, *p* < 0.001) and pointing (*z* = 15.08, *p* < 0.001) as compared to naming (*z* = 10.01, *p* < 0.001). Then, considering head-rotation extent, we documented the main effect of speaker position (*X*^2^ (1) = 90.06, *p* < 0.001), a main effect of listening condition (*X*^2^ (1) = 163.50, *p* < 0.001), a main effect of side (*X*^2^ (1) = 17.83, *p* < 0.001), three two-way interactions between speaker position and listening condition (*X*^2^ (1) = 70.14, *p* < 0.001) and side and listening condition (*X*^2^ (1) = 16.66, *p* < 0.001) and side and group (*X*^2^ (2) = 6.76, *p* = 0.03) and a three-way interaction between side, block and group (*X*^2^ (2) = 16.51, *p* < 0.001). These results suggested that participants increased their space explored in the altered listening condition, especially when sounds were emitted from the altered side (right) for reaching and pointing, and unaltered side (left) for naming. Considering the approaching index, we found a main effect of speaker position (*X*^2^ (1) = 30.67, *p* < 0.001) and listening condition (*X*^2^ (1) = 141.03, *p* < 0.001) and two two-way interactions between speaker position and listening condition (*X*^*2*^ (1) = 20.76, *p* < 0.001) and side and group (*X*^*2*^ (2) = 37.67, *p* < 0.001) and block and group (*X*^*2*^ (2) = 55.67, *p* < 0.001). Participants approached more toward the space of the speakers during the altered listening condition and particularly when the sounds were emitted by the periphery of the array. This behavior was implemented to a greater extent by the naming (*t* = 11.88, *p* < 0001) and the pointing group (*t* = 11.37, *p* < 0.001) than the reaching (*t* = 2.56, *p* = 0.01).

As expected, participants’ performance decreased when exposed to asymmetrical mild-moderate hearing impairment, especially on the ipsilateral side. The Pointing group’s sound localization error was higher if compared to naming and reaching groups. This result was unexpected. However, it is important to underline that this cannot be related to the different posture adopted by the pointing group, as documented for the binaural condition (block 1), because in block 2 the Pointing group implemented an approaching behavior similar to the one of the other two groups, and increased head movements during the altered condition (if compared with the binaural one). Furthermore, there is no evidence of difficulty in precisely controlling where to move the hand to point at the desired location, because in block 1 the error of the Pointing group did not differ from the other two groups.

### Sound localization improvements across repetition during simulated asymmetrical hearing loss as a function of instruction (naming, pointing, reaching)

To investigate if sound localization improved across block repetition during simulated asymmetrical hearing loss as a function of instruction, we focused on the second and the third block of the experiment (colored in red and orange in Figs. 2 and 4). Note that additional analyses comparing the third block and first block to test if the improved sound localization during a simulated asymmetrical hearing loss is comparable to binaural hearing as a function of instruction (naming, pointing, reaching) are reported in the Supplementary Materials. Note that participants’ response times decreased in block 3 (9.12 s) as compared to block 2 (10.6 s) revealing that participants need less amount of time to respond (*t*(41) = 3.82, p < 0.001).

#### Performance errors

We entered absolute in a GLME model with block, speaker position and group as fixed effect and participants as random effect. We documented a main effect of block (*X*^*2*^ (1) = 32.21, *p* < 0.001) and a main effect of speaker position (*X*^*2*^ (1) = 5.89, *p* = 0.02 and two-way interaction between block and group (*X*^*2*^ (2) = 52.28, *p* < 0.001) (Fig. 2A). Absolute error decreased during the block 3 as compared to the block 2. Furthermore, especially for the pointing group absolute error was higher for the speakers positioned in the side ipsilateral to the ear with the altered hearing experience. Interestingly, performance improvement (difference between block 3 and 2) was greater for pointing (*z* = 13.31, *p* < 0.001, b2 = 18.2° ± 16.1; b3 = 10.3° ± 11.7) and reaching (*z* = 14.64, *p* < 0.001, b2 = 12.7° ± 13.6; b3 = 6.0 ± 5.0) as compared to naming (*z* = 5.68, *p* < 0.001, b2 = 12.1° ± 14.0; b3 = 9.2° ± 11.7). Note that during block 3 the reaching group presented lower absolute error (6.0°) as compared to the pointing group (10.3°) although these values are not statistically different. Considering signed error, we found similar effects: a main effect of speaker position (*X*^*2*^ (1) = 15.47, *p* < 0.001), of group (*X*^*2*^ (2) = 7.60, *p* = 0.02) and two two-way interactions between speaker position and block (*X*^*2*^ (1) = 4.53, *p* = 0.03), and block and group (*X*^*2*^ (2) = 9.10, *p* = 0.01) and a three-way interaction between group, block and speaker position (*X*^*2*^ (2) = 12.60, *p* = 0.002). As shown in Fig. 2B, signed error decreased during the block 3 (orange) as compared to the block 2 (red) and particularly when sounds were emitted by the speakers positioned to the side ipsilateral to the ear with the altered hearing experience. Interestingly, the signed error reduction emerged selectively for the pointing group (*t* = 5.24, *p* < 0.001), but not for the reaching group (*t* = 1.79, *p* = 0.07) and the naming group (*t* = 1.34, *p* = 0.18). However, when we considered the decrement as a function of speakers position (expressed by the slopes in the figure and statistically by the three-way interaction), we documented that slope reduction during the block 3 as compared to the block 2 was higher for reaching (*t* = 5.79, *p* < 0.001) and pointing (t = 6.93, *p* < 0.001) as compared to naming group (*t* = 2.13, *p* = 0.03).

**Figure 5:**
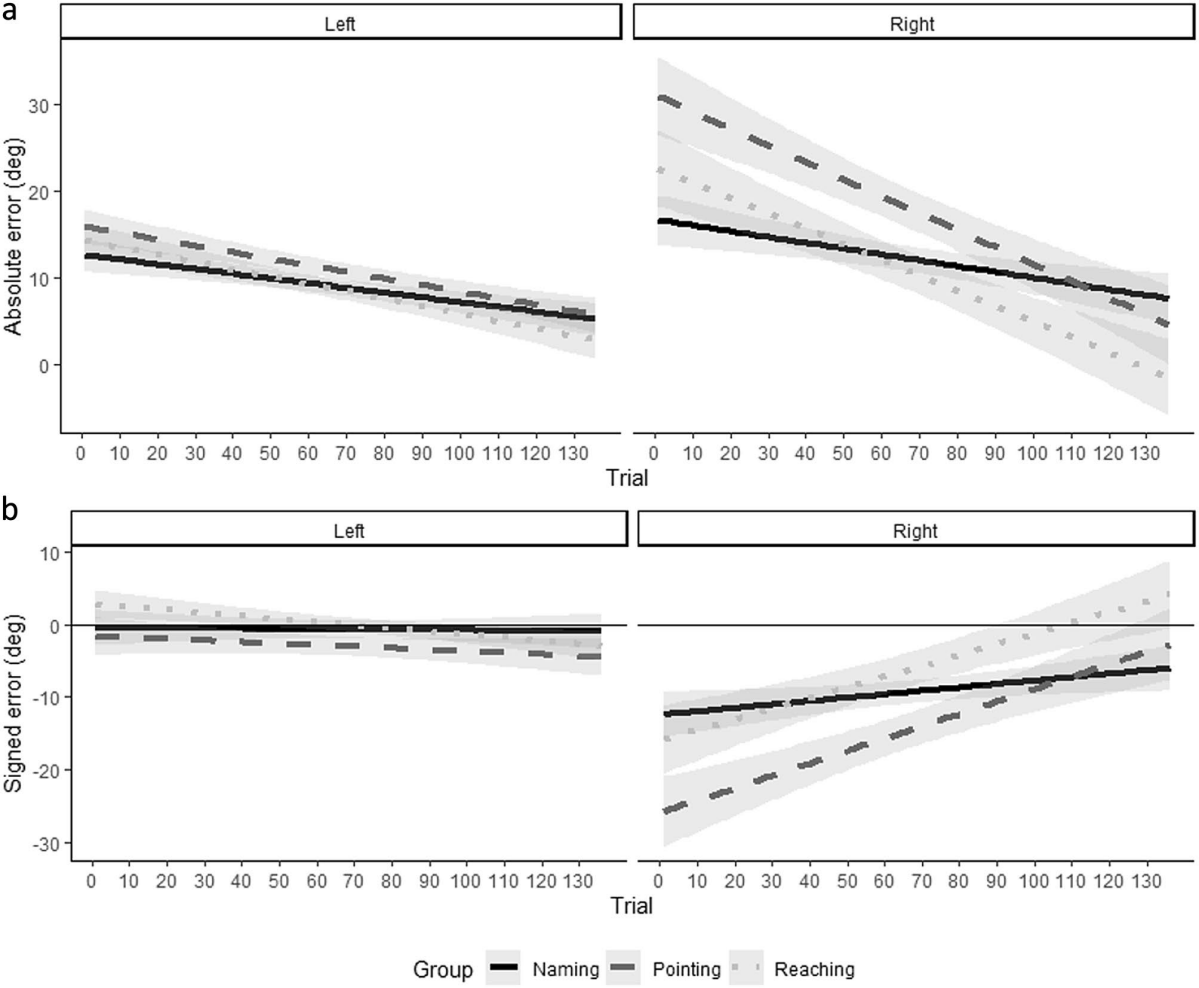
Performance across trial repetition during altered hearing blocks. Absolute error (**A**) and signed error (**B**) as a function of trials during the altered hearing blocks (x axis), side (left or right) and group (Naming: continue line; Pointing: dashed line, triangles; Reaching: points line). Linear regression (solid line), with 95% confidence intervals.

To study errors as a function of trial repetition during the altered listening condition, we further analyzed performance by entering them into three separate models and we considered side of sound, group and trial (we combined together block 2 and 3 and considered trials as a continuous variable from 1 to 136) as fixed effects and participant (intercept and slope) as random effects. Considering the absolute errors, we found a main effect of trial (*X*^*2*^ (1) = 12.55, *p* < 0.001), a main effect of side (*X*^*2*^ (1) = 47.06, *p* < 0.001), a two-way interaction between group and side (*X*^*2*^ (2) = 25.75, *p* < 0.001) and a three-way interaction between trail, side and group (*X*^*2*^ (2) = 8.36, *p* = 0.02). Absolute errors decreased as a function of trial repetition for each group and were higher for the right side for the pointing group. Interestingly, absolute error for the right side decreased across trials differently as a function of group (as revealed by slopes in Fig. 5A), but these differences did not emerge statistically by running a further analysis exclusively on the right side. Plus, we observed that the slopes describing trials’ trend differed between right and left side only for reaching (*z* = 4.05, *p* < 0.001) and pointing (*z* = 2.74, *p* = 0.006), but not for the naming group (*z* = 0.26, *p* = 0.80). Considering the signed errors, we found a main effect a main effect of side (*X*^*2*^ (1) = 82.28, *p* < 0.001), a two-way interaction between group and side (*X*^*2*^ (2) = 16.08, *p* < 0.001) and a three-way interaction between trail, side and group (*X*^*2*^ (2) = 20.85, *p* < 0.001). Signed error decrement was presented exclusively for the right side of the space and was higher for the pointing group. Interestingly, the three-way interaction suggested that signed error for the right side decreased across trials differently as a function of group (as revealed by slopes in Fig. 5B), but these differences did not emerge statistically by running a further analysis exclusively on the right side. Plus, we observed that the slopes describing trials’ trend differed between right and left side only for reaching (*z* = 7.24, *p* < 0.001) and pointing (*z* = 7.63, *p* = 0.006), but not for the naming group (*z* = 1.73, *p* = 0.06). To further document this effect on participants’ performance, we reported similar analyses on head movements in the SM.

#### Subjective judgments

We did not document any effect of the group on participants' rates of perceived effort during the task and their judgments about their performance (all *ps* > 0.42). We did not observe a significant decrement in the perceived effort reported during the block 3 (Friedman Test: *X*^*2*^ (1) = 3.19, *p* = 0.07), while participants reported to performed better during the block 3 as compared to the block 2 (Friedman Test: *X*^*2*^ (1) = 3.81, *p* = 0.05) (Fig. 3). We also analyzed the effect of the group on perceived improvements. We did not find any differences by performing a non-parametric Kruskal–Wallis Test (H(2)2.06, *p* = 0.36; naming: 4.1 ± 2.4; pointing: 4.9 ± 2.6; and reaching:5.4 ± 2.1) (Fig. 3C).

#### Head-related behavior

We entered number of reversals in a GLM model (family Poisson) with speaker position, block, side as fixed effects and participant (slope and intercept) as random effects. We found three two-way interactions between side and block (*X*^*2*^ (1) = 6.27, *p* = 0.01) and block and group (*X*^*2*^ (2) = 16.02, *p* < 0.001) and side and group (*X*^*2*^ (2) = 11.50, *p* = 0.003). These results suggested that the number of movements are higher for the right side as compared to the left side only for the pointing group (*z* = 3.62, *p* < 0.001), but not for the other two groups (*ps* > 0.47). Plus, while the naming group did not change the number of movements between the two blocks (*z* = 0.08, *p* = 0.94), the reaching and the pointing group decreased their head movement in the block 3 as compared to the block 2 (reaching: *z* = 2.31, *p* = 0.02; pointing: *z* = 5.70, *p* < 0.001). The decrement was observed when sounds were emitted by the speakers in the right space (*z* = 4.98, *p* < 0.001), but not in the left space (*z* = 1.73, *p* = 0.08). Considering head-rotation extent, we found a main effect of speaker position (*X*^*2*^ (1) = 25.10, *p* < 0.001), a main effect of side (*X*^*2*^ (1) = 46.82, *p* < 0.001), four two-way interactions between side and speaker (*X*^*2*^ (1) = 4.57, *p* = 0.03) and side and block (*X*^*2*^ (1) = 4.01, *p* = 0.04), side and group (*X*^*2*^ (2) = 33.37, *p* < 0.001) and block and group (*X*^*2*^ (2) = 18.51, *p* < 0.001) and a three-way interaction between speaker, block and group (*X*^*2*^ (2) = 7.07, *p* = 0.03). These results suggest that space explored decreased in block 3 as compared to the 2 and especially for the Reaching group (*t* = 4.41, *p* < 0.001; ps for other groups > 0.21). Plus, we observed that the effect of speaker position (slope) was more pronounced in block 2 as compared to block 3 only for the reaching group (*t* = 2.34, *p* = 0.02), but not for the other groups (*ps* > 0.16). By analyzing the approaching index, we found a main effect of speaker position (*X*^*2*^ (1) = 14.17, *p* < 0.001) and side (*X*^*2*^ (1) = 7.85, *p* = 0.005) and two two-way interaction between side and group (*X*^*2*^ (2) = 90.94, *p* < 0.001) and block and group (*X*^*2*^ (2) = 10.17, *p* = 0.006) and a three-way interaction between side, block and group (*X*^*2*^ (2) = 8.95, *p* = 0.01). Participants of reaching and pointing group increased their approaching movements in the block 3 as compared to block 2 especially for the left side for the reaching group (*t* = 3.50, *p* < 0.001), but not for the other groups (*both p*s > 0.06) and for the right side for the pointing group (*t* = 4.59, *p* < 0.001), but not for the other groups (*both p*s > 0.79) (Fig. 4).

### After-effect in virtual binaural conditions after having experienced simulated asymmetrical hearing loss, as a function of instruction (naming, pointing, reaching)

To investigate if we could observe any after-effect when performing sound localization in binaural condition after having experienced simulated asymmetrical hearing loss as a function of instruction, we focused on the experiment’s first and fourth block (colored in black and grey in Figs. 2 and 4). Note that this analysis was explorative and should be influenced by the fact that in block 1 participants experienced feedback about their performance while this did not happen in block 4. However, we controlled this aspect by entering absolute error a GLME model (family Poisson) with trial, speaker position, and group as fixed effects and participant (intercept and slope) as random effects and we observed that despite performance improved across trial (*X*^*2*^ (1) = 7.52.03, *p* = 0.006), the improvement did not change as a function of blocks (interaction between block and trial, *X*^*2*^ (1) = 1.04, *p* = 0.31).

#### Performance errors

We entered absolute error a GLME model (family Poisson) with block, speaker position and group as fixed effect and participant (intercept and slope) as random effects. We found a main effect of block (*X*^*2*^ (1) = 4.86, *p* = 0.35) and a two-way interaction between speaker position and block (*X*^*2*^ (1) = 3.78, *p* = 0.05). These results suggested that in block 4 the absolute error reduced more for the speaker positioned to the left. Analysis on signed error revealed a main effect of speaker position (*X*^*2*^ (1) = 14.03, *p* < 0.001), a main effect of block (*X*^*2*^ (1) = 27.44, *p* < 0.001), a two-way interaction between speaker position and group (*X*^*2*^ (2) = 6.19, *p* = 0.05) and a three-way interaction between group, block and speaker position (*X*^*2*^ (2) = 9.22, *p* = 0.01). Interestingly, the effect of speaker position presented in the block 1 decreased in the block 4 exclusively for the reaching group (*t* = 2.85, *p* = 0.004), but not for naming (*t* = 1.74, *p* = 0.08) and pointing group (*t* = 1.30, *p* = 0.19) (Fig. 2).

#### Subjective judgments

We did not document any effect of group (all *ps* > 0.12) on perceived effort during the task and their judgments about their performance. We did not observe any differences between block 1 and 4 both for perceived effort (Friedman Test: *X*^*2*^ (1) = 0.21, *p* = 0.65) and judgments about their performance (Friedman Test: *X*^*2*^ (1) = 1.38, *p* = 0.24) (Fig. 3).

#### Head-related behavior

We entered number of reversals in a GLME model (family Poisson) with speaker position, block, side as fixed effects and participant (slope and intercept) as random effects. We observed a main effect of block (*X*^*2*^ (1) = 17.97, *p* < 0.001), a two-way interaction between group and block (*X*^*2*^ (2) = 147.89, *p* < 0.001). For the naming group, the number of head movement decreased in the block 4 as compared to 1 (*z* = 4.06, *p* < 0.001), for the pointing group they did not change (*z* = 0.47, *p* = 0.64), while for the reaching group the head movements increased in the block 4 as compared to the block 1 (*z* = 12.48, *p* < 0.001). Then, by entering head-rotation extent in a similar analysis, we found a main effect of speaker position (*X*^*2*^ (1) = 79.01, *p* < 0.001), two two-way interactions between speaker position and block (*X*^*2*^ (1) = 8.92, *p* = 0.003) and group and block (*X*^*2*^ (2) = 21.04, *p* < 0.001). These results suggested that in block 4 the space explored was higher as compared to block 1 specifically when sounds were emitted by peripheral speakers. Only the reaching group increased their space explored in block 4 as compared to block 1 (*t* = 4.87, *p* < 0.001), but not the other two groups (all *ps* > 0.13). Considering the approaching index, we found a main effect of speaker position (*X*^*2*^ (1) = 36.75, *p* < 0.001) and block (*X*^*2*^ (1) = 38.20, *p* < 0.001) and a two-way interaction between side and group (*X*^*2*^ (2) = 29.29, *p* < 0.001) and block and group (*X*^*2*^ (2) = 9.41, *p* = 0.009) and a three-way interaction between speaker position, block and group (*X*^*2*^ (2) = 16.89, *p* < 0.001). Participants approached more toward the space of the speakers during block 4 as compared to block 1. The reaching group increased this behavior particularly when the sounds were emitted by the central speaker (i.e. slopes differed, *t* = 4.76, *p* < 0.001), while the pointing group when they were emitted by the peripheral ones (i.e. slopes differed, *t* = 4.14, *p* < 0.001). On the contrary, the naming group increased this behavior irrespectively of speaker position (i.e. slopes did not differ, *t* = 0.57, *p* = 0.57) (Fig. 4).

### Relations between performance, improvement rating, and head-related behavior.

In order to gain insight into the relation between the improvement itself and the internal representation of improvement that each individual has created by doing the task and how this relationship is influenced by the different interactions with sound sources (i.e. naming, reaching, and pointing), we calculated the difference between performance during the block 3 and 2 to obtain three indices of improvement: absolute errors decrement and % of correct responses increment. We correlated these two indices with improvement rated by participants. We found a significant correlation between perceived improvement and % of correct responses increment (Spearman’s rho: 0.50, p < 0.001), while the correlation between perceived improvement and absolute errors decrement did not reach the significance (Spearman’s rho: 0.26, p = 0.09). The significant correlation observed revealed that participants' assessment of their improvement was based specifically on the % of correct responses and not on the extent of errors, which was instead captured by absolute error. We also correlated the measure of performance improvement with the measure of head-related behavior changes. We observed that the more participants increase their approaching behavior, the more their % of correct responses increased (Spearman’s rho = -0.46 p = 0.002), but we did not observe any correlations between other parameters (*p* > 0.14). Note that if we correlated these values by considering each group separately, correlations remained significant only for the pointing group and not for both naming and reaching groups.

Furthermore, we studied the relation between head-related behavior and performance during the altered hearing experience (average of block 2 and 3) in order to investigate whether the head-related behavior implemented by the participants were functional to their performance and if this relation could differ as a function of the instruction. We found that the more participants implemented approaching movements the better their performance was: the greater the approaching index the higher % of correct response (Spearman’s rho = -0.53, *p* < 0.001), the lower absolute (Spearman’s rho = 0.50, *p* < 0.001) and signed error (Spearman’s rho = 0.33, *p* = 0.04). Similar results were observed also by correlating the number of head movements, but not the extent of head rotation (all *ps* > 0.21). The higher number of reversal participants implemented, the higher % of corrected responses (Spearman’s rho = 0.40, *p* = 0.008) was and the lower absolute error was (Spearman’s rho = − 0.39, *p* = 0.01). These relationships suggest a general benefit of implementing head movements in reducing sound localization errors. Note that if we correlated these values by considering each group separately, correlations remained significant only for the naming group (Spearman’s rho = 0.62, *p* = 0.02 and Spearman’s rho = − 0.62, *p* = 0.02, respectively) and not for both pointing and reaching groups (all ps > 0.06).

### Questionnaire

At the end of the experimental session, we proposed a questionnaire to investigate the general experience of participants with the task and the VR equipment. We adopted a questionnaire that we have already proposed in a previous paper^14^ which was inspired by the User Engagement Scale (UES) Questionnaire^45^. We tested group effect on the 3 subscales of agency (comprising two items, Cronbach’s alpha: 0.56), focus of attention (comprising seven items, Cronbach’s alpha: 0.74), and satisfaction (comprising seven items, Cronbach’s alpha: 0.78). We did not find any effect of the group either for cumulative indices (all *ps* > 0.34) or for single items (all *ps* > 0.15) (see Supplementary Results).

## Discussion

In the present study, we explored the contribution of reaching to sound sources when adapting to altered auditory cues by comparing three groups of normal-hearing participants while performing a sound localization task in both normal and altered listening conditions. We observed that all groups improved across altered listening blocks, but the extent of the improvement was higher for Reaching and Pointing as compared to the Naming group. Crucially, the reaching group reached a lower error rate when exposed to asymmetrical mild-moderate hearing impairment. Finally, while performance in the unaltered blocks (first and last) was comparable, only the reaching group continued to exhibit a head behavior similar to those developed during the altered blocks (second and third), corroborating the previous observed relationship between reaching to sounds task and head movements.

### The effectiveness of reaching to sounds

#### Reaching is more than just involving hand in the response method

Albeit the audio-visual feedback was exactly the same, our analysis suggested that performance improvement is greater for reaching and pointing groups as compared to the naming group. This result suggests that involving the hand in the task may subtend different and more effective learning processing. Moreover, the reaching group is clearly distinguished from pointing and naming groups because participants’ errors (specifically for the ipsilateral side of the alteration) are clearly lower as compared to the ones made by the other two groups (Fig. 2). Coherently, the difference between the performance in the altered hearing condition (block 3) and the binaural performance (block 1) is smaller for this group as compared to the other two (see SM). Considering head movements, we documented that participants of the reaching and pointing group (but not of the naming one) increased their approaching behavior from block 2 to block 3 meaning that they increased their head-related strategies in the altered hearing condition. This observation could reveal the potential of reaching and pointing in triggering the implementation of behavioral strategy during altered listening. Although it is important to note that even the naming group was able to change behavioral strategy in block 2 relative to block 1 and then maintained this approach in block 3. However, the increasing approaching strategies occurred in block 3 relative to block 2 could have contributed to decreasing participants’ error as it permitted to compare the differences of sound intensity reaching the ear as a function of head position and exploit this computation to localize the sources. On the contrary, the extent of head rotation decreased from block 2 to 3 indicating an adjustment of the use of this movement across the altered hearing exposure. Interestingly, the extent of head rotation decreased specifically for the central speaker exclusively for the reaching group meaning that this group may have learned to explore the space in an increasingly functional manner across time.

As reported above, reaching groups lead to a greater improvement as compared to both pointing and naming. This finding corroborates the rationale behind the reaching-to-sounds’ benefit hypothesis we argued in our previous work claiming that the action of reaching toward a certain position in the space requires coordinating different effectors (eyes, head, hand) into a common reference frame^27^ resulting in a more stable or salient^51^ spatial coding of sound source location. Our results support the hypothesis that the key feature of 'reaching-to-sounds' is the physical movement (action) of the body (the hand) toward the target space and not the mere involving the hand. This finding represents a novel insight and marks a crucial initial step towards documenting the unique characteristics of the 'reaching-to-sounds' effect. This type of observation also calls into play the concept of peripersonal space. Acting on a given object involves including that object in a multisensory and attentive spatial representation with special characteristics^28^. Planning to reach an object is able to strengthen multisensory coding of this object and thus it may favor the process of associating spatial position and auditory cues simultaneously experienced^30^. This does not happen with pointing as there is no direct action towards the sound. In fact, note that in creating the pointing task we were careful not to give the laser characteristics of a tool, which could have been perceived as an extension of one's body (e.g. vibration in correspondence with the collision^52^). Note that, despite the fact that the space usually attributed to peripersonal space is plausibly involved in the reaching condition as the target sources were positioned at 55 cm (see for instance^53^), in this study we did not collect a typical measure of peripersonal space by adopting a classical multisensory integration task (e.g. audio-tactile interaction paradigms, ^54,55^). Thus, in order to deepen the role of peripersonal space in this type of interaction with sound sources, future studies should also include this task.

Evidence of the efficacy of audio-motor training has been demonstrated in recent studies that use audio-motor feedback in conjunction with an innovative device (i.e. an audio bracelet). These studies highlight the potential of sensory feedback provided by the device to establish a connection between auditory and motor signals, thereby linking the body with external representations of the environment. Notably, the use of this audio bracelet has been investigated and found to have a positive impact on spatial perception in both sighted and blind individuals, affecting spatial awareness of the upper body as well as the legs^13,56–60^. In comparison to the type of motor interaction proposed in our study (i.e., reaching or pointing), the interaction involved in the use of this device presents some differences. In our study, it is not the participant who moves the sound with their body. The hand and sound only coincide at the same location in the reaching condition, specifically when a response is provided (i.e., the hand reaches the source). However, both scenarios may reflect similar processes of sensorimotor association related to action and contribute to documenting the influence of action on spatial perception^61,62^. These findings, therefore, call for further investigation into the link between action and spatial perception. In addition, they could prompt further research on the links between action and auditory processing at the behavioral and neural level. These interactions are largely pervasive and lead to systematic multisensory-motor associations in everyday life (i.e., environmental statistics). Consider for instance pressing keys on a keyboard while typing, or reaching towards a plastic bottle that squeezes with noise during our action. Recent fMRI studies^63^ indicate that action planning can modulate primary sensory areas. Yet, it remains to be ascertained to what extent this can also contribute to strengthening the representation of auditory cues, or contribute to consolidate novel associations between auditory cues and spatial coordinates of the sound source. Future research could delve deeper into these topics by taking advantage of techniques such as fMRI, EEG, muscle contraction and similar.

A further interpretation of the reaching-to-sounds’ benefit, which is not mutually exclusive with the one described in the above paragraphs, could be related to the power of motor action to facilitate categorization processes. Categorization processes consist in the recognizing of a set of elements and rules to define objects ^64^. Some researchers stressed the importance of considering objects' perception intrinsically related to the pattern of action they evoke (see the sensorimotor contingencies proposed by O’Regan and Noe^65^. According to this idea, the knowledge of the external objects coincides with the set of percepts resulting from the active interaction with them^66–68^. If people use precise patterns of objected-related action to categorize objects, implementing repetitive actions toward a certain object may facilitate its categorization. Following this reasoning, continuously implementing reaching movements toward 17 possible sources, as performed by the reaching group, could help to create 17 distinct associated percepts and thus favor sound localization learning processes.

#### Reaching to sound and head movements

Head movements showed changes in all groups as the listening condition became impaired. These alterations in head movements can be seen as spontaneous strategies that individuals employ in response to the increased demands of the task. This suggests that participants adapt their head movements as a way to cope with the difficulties posed by the impaired listening condition^69^. In addition to the elements described in previous paragraphs, a further peculiarity of the reaching to sound is its link with head-related strategies implementation. In the present study, we did not observe a clear relation between the improvement in terms of performance and the increase in the use of head-related strategies, which was instead documented in a previous experiment^14^. The head movement patterns observed when testing participants in altered listening condition did not suggest any peculiarity in using head-related behavioral strategies proper of the reaching group compared to naming and pointing groups (see 4.1.3). However, in this study, we documented that only the reaching group continued to use the behavioral strategies they implemented during the altered condition, in the normal listening condition (4.1.4). This after-effect clearly suggests that head movements were triggered by this type of active interaction with the sound source more than the other two we tested. As far as we know, this is the first time that this “strategies’ after-effect” was observed. However, it remains to establish what is the prominent aspect able to capture the reaching to sound benefit. Our findings are not sufficient to lean towards one aspect over another and the most plausible hypothesis at the moment is that all the aspects mentioned contribute to describe this effect. Further studies are needed to disambiguate the direct effect of reaching to sounds training on sound localization abilities from the effect mediated by head movements. A possible strategy to address this new experimental question could consist of manipulating the possibility of moving the head, even if this would necessarily change the experiment having to consider a smaller space that has to be visible even with the head still.

### On the consequences of adapting to simulated asymmetrical hearing loss

We did not find any after-effect by measuring localization errors, but we observed an interesting after-effect of head-related behavior: exclusively the reaching group continued to move their heads during the final block as in the previous altered blocks. Considering the general themes of adaptation and after-effects in the spatial hearing research field, some studies have tried to measure the permanence of training with the aim of seeing if the ability to localize in altered conditions had been maintained in a subsequent moment in which participants were tested in the same altered conditions^23,48,49^. On the contrary other studies focused properly on the after-effect as we intended in the present study, but they focused on sound movement perception without investigating the ability to localize stationary sounds^50^. Thus, as we declared in the Introduction, it was hard to have a clear prediction based on the literature. However, a stable change in the acoustic space processing can be observed looking at the absence of alterations in the performance resulting from compensatory response behaviors with respect to any systematic adjustments introduced by the participants during the altered listening condition (i.e. after-effects) when performed the task in block 4. All groups were able to perform in block 4 as (or better than) they did in block 1 indicating that they were all able to combine and weigh the auditory cues without being anchored to the way in which they had combined them during the altered hearing experience. However, we documented a curious and unexpected phenomenon by analyzing head-related behavior: all the indices we considered clearly indicate (see Fig. 4) that exclusively the Reaching group re-proposed in the last block the same behavioral strategies used in the altered blocks and they clearly differed from the strategies adopted in the block 1. The head-related behavior implemented during the altered listening blocks (2 and 3) was implemented also in the block 4 in which the listening condition was instead binaural hearing meaning that only the reaching group continued to use the behavioral strategy learned and implemented during the simulated asymmetrical hearing loss even if the listening condition changed. We interpreted these results as a marker of the previous observed relationship between reaching to sounds task and head movements (see 4.2 for further discussion about that).

### Acoustic virtual reality is a reliable tool to study sound localization re-learning

An additional aim of the present work was to test whether we could observe improvement in localizing sound sources even by adopting a fully-virtual approach. As expected, the present results are coherent with our previous work. In this experiment, the group was able to reduce their absolute error in azimuth from 12.7° (mean Block 2) to 6° (mean Block 3). Note that one block comprised 68 trials. In the work of Valzolgher and colleagues ^14^ the reaching group was tested by adopting a method that allows the use of a single real speaker which was moved by the experimenter while participants are immersed in a virtual visual scenario. It had an absolute error of 12.2° (mean Block 2) that reduced to 7.2° (mean Block 5) after performing 5 blocks of 51 trials each indicating that the two approaches are comparable. This finding represents an intriguing novelty from a practical standpoint, with the potential to allow the development of a reaching-to-sound training that relies entirely on portable equipment, thus eliminating the need for an external operator. Adopting a fully-virtual approach, even reaching-to-sound training could be implemented outside the laboratory. Moreover, virtual audio allows much more flexibility. For example, in this test, we decided to go for a certain number of discrete sources, but this can be increased as much as we want without additional cost. Furthermore, we can also simulate different acoustics conditions (e.g. larger or smaller rooms, background noises…). Future studies should test its efficacy and feasibility in the field of remote rehabilitation.

### Future directions and limitations

Testing a group of participants who performed sound localization by pointing to the source gave us the possibility to test directly the power of reaching sound sources’ positions net of hand involvement in the response. However, this condition resulted in an unexpected finding when testing participants in altered listening conditions: the pointing group’s error in sound localization was higher as compared to naming and reaching groups. As discussed above (see 4.1.1 and 4.1.2), the decrease of error could not be related exclusively to the change in body posture assumed by the participants when directing the laser toward the sources nor to difficulties in controlling were direct the hand, as in the task we proposed participants were asked to select from a discrete number of clearly visible speakers. Nevertheless, this aspect could constitute a limitation of the comparison between reaching and pointing conditions in this particular task. Future studies could investigate further this comparison even by extending the space occupied by the speaker in terms of distance to make the pointing action more comfortable. Plus, concerning the possibility of manipulating the space as a result of using tools^70,71^, future lines of research could test the training paradigm aiming at fostering improvements in different portions of the space by using tools and thus expanding the comparison between reaching and pointing condition. A further aspect to be explored concerns the relationship between the laterality of the effector (i.e. manual preference) and laterality of the auditory alteration (right or left ear). Further investigating these aspects in future studies could contribute to document the effect of reaching to sounds on sound localization^72^.

Another aspect highlighted by this research concerns the auditory after-effect. A clear methodological limitation of our experimental design is the differences between the two blocks (1 and 4). They differ in two aspects: the timing (i.e., before and after the exposure to altered auditory cues) and the presence of audio-visual feedback (i.e. available during the first block, but not during the second one). This discrepancy may potentially act as a confounding factor, warranting further investigations in future studies to rigorously examine this effect. Introducing the study of the after-effect could reveal the consequences of implementing specific response strategies or biases, which were not observed in the present study. Additionally, it may shed light on the implications of exposure to altered auditory stimulation on the person's spontaneous behavior and strategic movements implemented by people. Notably, intriguing head movement patterns were observed in this study. Future research may delve deeper into these observations to gain a more comprehensive understanding of the subtending mechanisms.

A further limitation is the choice of an experimental design comparing three different groups of 14 participants. In order to investigate the differences between these three ways of interacting with sound sources, the choice of a between-groups experimental design was forced by the impossibility to engage all participants in all conditions, as this would have resulted in training/order effects which would have been difficult to deal with. However, the prominent inter-individual variability reduced the power of our analysis and prevented us from adopting more flexible statistical mixed models, which could have given us clearer and more significant results.

Finally, a further interesting line of research in this field consists in deepening the role of metacognitive evaluation while performing sound localization (see for instance studies in hearing in noise^73,74^). Although this theme was not the core of the present study, here we made a first exploratory step in this direction by measuring participants’ perceived effort and their judgment about their performance and improvement (see Fig. 3). We documented that both perceived effort and judgment about one’s own performance were modulated by the listening condition. However, we did not find any effect of instruction neither on these parameters nor on improvement judgment. Nevertheless, we think that metacognitive evaluations may be linked bidirectionally both to performance and behavior and future studies should investigate these aspects more systematically.

### Supplementary Information

Supplementary Information 1.

Supplementary Video 1.

Supplementary Video 2.

Supplementary Information 2.

## Data Availability

The datasets generated and analyzed during the current study are available in OSF repository, and retrieved from osf.io/4pykg.
