# Supplementary material for: Spatial hearing training in virtual reality with simulated asymmetric hearing loss
Source: Sci Rep. 2024 Jan 30;14:2469. doi: 10.1038/s41598-024-51892-0 (PMC10827792; doi:10.1038/s41598-024-51892-0)
Supplement: Supplementary file 1 — Supplementary Information 1. [file 41598_2024_51892_MOESM1_ESM.docx]

**Captions for supplementary videos 1 and 2**

Supplementary video 1. **Pointing task.** Trials examples of the pointing task. Participants performed a sound localization task and were instructed to point (using the red laser they oriented by using the controller held in their hands) the sound sources to localize them.

Supplementary video 2. **Reaching task.** Trials examples of the reaching task. Participants performed a sound localization task and were instructed to reach (using the visible controller held in their hands) the sound sources to localize them.
