# Supplementary material for: Spatial hearing training in virtual reality with simulated asymmetric hearing loss
Source: Sci Rep. 2024 Jan 30;14:2469. doi: 10.1038/s41598-024-51892-0 (PMC10827792; doi:10.1038/s41598-024-51892-0)
Supplement: Supplementary file 4 — Supplementary Information 2. [file 41598_2024_51892_MOESM4_ESM.pdf]

Spatial hearing training in virtual reality with simulated asymmetric hearing loss

Chiara Valzolgher[1], Sara Capra[1], Kevin Sum[2] , Livio Finos[3], Francesco Pavani[1,4,5*] & Lorenzo Picinali[2*]

1. Center for Mind/Brain Sciences (CIMeC), University of Trento, Italy
2. Audio Experience Design (www.axdesign.co.uk), Imperial College London, UK
3. Department of Statistical Sciences, University of Padova, Italy
4. Department of Psychology and Cognitive Sciences (DiPSCo), University of Trento, Italy
5.Centro Interuniversitario di Ricerca "Cognizione, Linguaggio e Sordità" (CIRCLeS), Trento, Italy

Keywords: Sound localization, virtual reality, reaching to sounds, altered listening situation, simulated mild-moderate unilateral hearing loss.

*These authors contributed equally to this work.

Summary

This Supplementary Materials document includes additional results which could not be directly included in the paper:

1. Further analysis on performance errors: % of correct responses
2. Further experimental question to investigate participants improvement: Could the improved sound localization during simulated asymmetrical hearing loss be comparable to binaural hearing as a function of instruction (naming, pointing, reaching)?
3. Does sound localization improve across block repetition during simulated asymmetrical hearing loss as a function of instruction (naming, pointing, reaching)? – Head movements
4. Questionnaire

Supplementary Results

1. Further analysis on performance errors: % of correct responses

To document the performance, we measured the % of correct responses (Figure S1 and S2).

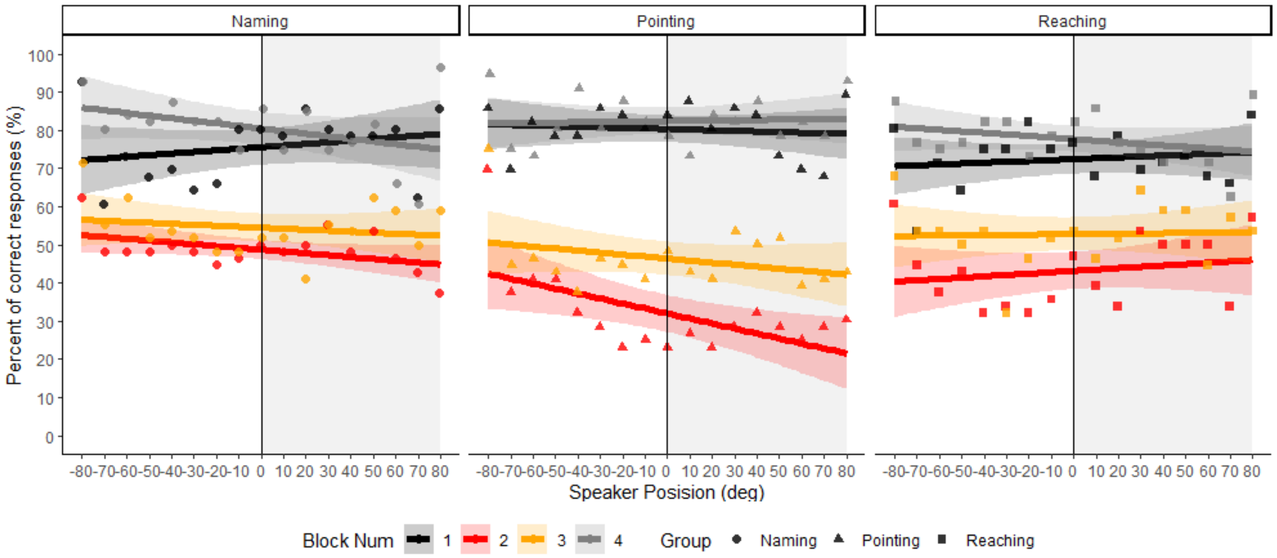

Figure S1. Percent of correct responses across the four blocks (1: black; 2: red; 3: orange and 4: grey) and as a function of speaker position (x axis) and group (Naming: to the left, points; Pointing: center of the figure, triangles; Reaching: to the right, squares). Linear regression (solid line), with 95% confidence intervals.

1.1 Sound localization during virtual binaural hearing as a function of instruction (naming, pointing, reaching)

We entered participants' response correctness (encoded as 0 or 1) during the first block (black) in a GLME model (family binomial) with speaker position (considered as a continuous variable) and group (naming, pointing or reaching) as fixed effects. To account for the variability related to individual participants, we also included participant (intercept and slope) as random effects. We did not find any effects of group or speaker position (all *ps* > 0.33).

1.2. Sound localization when altering hearing experience by simulating asymmetrical hearing loss as a function of instruction (naming, pointing, reaching)

We entered response correctness in a GLME model (family binomial) with listening condition (binaural-block1 and altered listening-block 2), speaker position (encoded as if all participants listened with their right ear in an altered manner) and group as fixed effect and participants (slope and intercepts) as random effects. We found a main effect of listening condition (X^2 (1) = 181.56, $p < 0.001$) and two two-way interactions between speaker position and listening condition (X^2 (1) = 7.51, $p = 0.006$) and listening condition and group (X^2 (2) = 48.62, $p < 0.001$) and a three-way interaction between listening condition, group and speaker position (X^2 (2) = 5.91, $p = 0.05$). These results suggested that percent of correct responses decreased during the altered hearing (block 2) as compared to the binaural hearing (block 1) and particularly for the speakers positioned in the side ipsilateral to the ear with the altered hearing experience. Interestingly, performance decrement was more pronounced for the pointing group ($z = 20.46$, $p < 0.001$) as compared to the naming ($z = 13.47$, $p < 0.001$) and the reaching group ($z = 13.33$, $p < 0.001$). The three-way interaction revealed that the decrement of correct responses is particularly evidence for the speakers positioned in the side ipsilateral to the ear (slope) with the altered hearing experience for the pointing ($z = 2.70$, $p = 0.007$) and naming group ($z = 2.74$, $p = 0.006$), but not for the reaching group ($z = 0.01$, $p = 0.99$).

1.3 Sound localization across block repetition during simulated asymmetrical hearing loss as a function of instruction (naming, pointing, reaching)

We entered response correctness in a GLME model (family binomial) with block (block 2, block 3), speaker position and group as fixed effect and participant (slope and intercept) as random effects. We found a main effect of block (X^2 (1) = 10.75, p = 0.001) and two interactions between speaker position and group (X^2 (2) = 10.13, p = 0.006) and block and group (X^2 (2) = 7.21, p = 0.03) (Figure 2A). Percent of correct responses increased during block 3 as compared to block 2. Furthermore, especially for the pointing group the percent of correct responses was lower for the speakers positioned in the side ipsilateral to the ear with the altered hearing experience. Interestingly, performance improvement (difference between block 3 and 2) was slightly greater for pointing (z = 7.20, p < 0.001, b2 = 32%±30; b3 =46%±33) as compared to naming (z = 3.28, p < 0.001, b2 =49%±37; b3 =55%38) and reaching (z = 4.50, p < 0.001, b2 = 43%30; b3 = 53%31), despite the fact that for the pointing group the % of errors was numerically higher as compared to the other groups.

To investigate the improvement during sound localization task, we further analysed errors dependent variables by entering them into three separate GLME models and we considered side of sound (left, right; encoded as if all participants listened with their right ear in an altered manner), group and trial (we combined together block 2 and 3 and considered trials as a continuous variable from 1 to 136) as fixed effects and participant (intercept and slope) as random effects. We found a main effect of trial (X^2 (1) = 5.15, p = 0.02) and a two-way interaction between group and side (X^2 (2) = 11.71, p = 0.003) revealing that correct responses increased across trial repetition and that, specifically for the right side, the pointing group performed a lower percent of correct responses. Considering the absolute errors, we found a main effect of trial (X^2 (1) = 12.55, p < 0.001), a main effect of side (X^2 (1) = 47.06, p < 0.001), a two-way interaction between group and side (X^2 (2) = 25.75, p < 0.001) and a three-way interaction between trail, side and group (X^2 (2) = 8.36, p = 0.02).

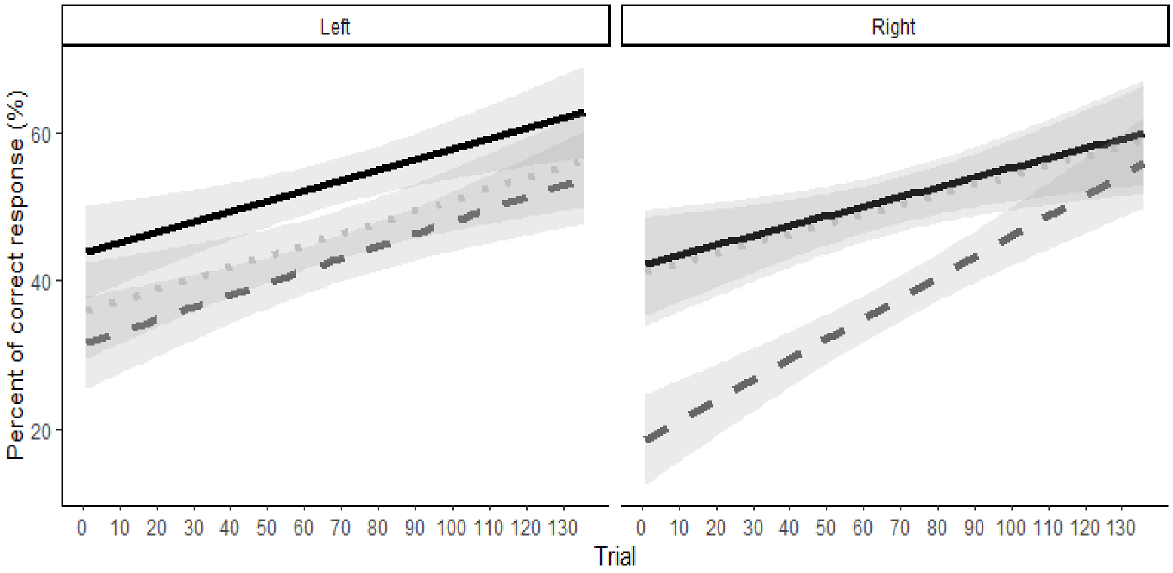

Figure S2. Percent of correct responses across trials during the altered hearing blocks (x axis), side (left or right) and group (Naming: continue line; Pointing: dashed line, triangles; Reaching: points line). Linear regression (solid line), with 95% confidence intervals.

1.4 After-effect in virtual binaural conditions after having experienced simulated asymmetrical hearing loss, as a function of instruction (naming, pointing, reaching)

We entered response correctness in a GLME model (family binomial) with block (block 1, block 4), speaker position and group as fixed effect and participant (slope and intercept) as random effects. We found a main effect of block (X^2 (1) = 9.23, p = 0.001) and a two-way interaction between speaker position and block (X^2 (1)

= 11.76, $p = 0.03$) and a three-way interaction between block, group and speaker position (X^2 (2) = 8.53, p = 0.01) (Figure 2A). These results suggested that percent of correct responses increased in the block 4 and this increment was greater for the left side (slope) for the naming group ($z = 3.43$, $p < 0.001$), but not for reaching ($z = 1.83$, $p = 0.07$) or pointing group ($z = 0.65$, $p = 0.51$).

2. Further experimental question to investigate participants improvement: Could the improved sound localization during simulated asymmetrical hearing loss be comparable to binaural hearing as a function of instruction (naming, pointing, reaching)?

We compared performance during the block 3 (coloured in orange) with the ones obtained during binaural hearing (block 1, coloured in black). The rationale was that the more the improvement we observed, the smallest the differences between performance during block 3 and block 1 should be.

2.1. Performance

We entered response correctness (encoded as 0 or 1) in a GLME model (family binomial) with block (block 1, block 3), speaker position and group as fixed effect. To account for the variability related to individual participants, we also included participant (slope and intercept) as random effects. We found a main effect of block (X^2 (1) = 121.82, $p = 0.001$), a main effect of speaker position (X^2 (1) = 3.96, $p = 0.05$), two two-way interactions between speaker position and block (X^2 (1) = 6.14, $p = 0.01$) and block and group (X^2 (2) = 28.68, $p < 0.001$) (Figure 2A). These results are in line to the ones previously described. Plus, they suggested that percent of correct responses differed between block 3 and block 1 with a greater extent for the pointing (z = 15.57, $p < 0.001$; b1: 80%±25; b3: 46%±33) and for naming ($z = 11.04$, $p < 0.001$; b1: 76%±30; b3: 55%±38) and with a smaller extent for the reaching group ($z = 9.27$, $p < 0.001$; b1: 72%±30; b3: 53%±31). We entered absolute error a similar GLME model and found a main effect of block (X^2 (1) = 268.07, $p < 0.001$), two two-way interaction between speaker position and block (X^2 (1) = 13.55, $p < 0.001$) and block and group (X^2 (2) = 65.79, $p < 0.001$). They suggested that absolute errors differed between block 3 and block 1 with a greater extent for the pointing ($z = 19.62$, $p < 0.001$; b1: 2.4°±3.7; b3: 10.3°±11.7) and for naming ($z = 16.37$, $p < 0.001$; b1: 3.0°±4.6; b3: 9.2°±11.7) and with a smaller extent for the reaching group ($z = 8.89$, $p < 0.001$; b1: 3.2°±3.7; b3: 6.0°±5.0). Furthermore, we observed a three-way interaction between block, speaker position and group (X^2 (2) = 6.15, $p = 0.05$) revealing that the effect of speaker position (represented by slopes in Figure 2B) did not differed between block 1 and block 3 only for the reaching group ($z = 0.67$, $p = 0.50$), while it differed for pointing ($z = 3.71$, $p < 0.001$) and naming group ($z = 3.68$, $p < 0.001$). Furthermore, when considering signed error, we documented a main effect of of block (X^2 (1) = 58.44, $p < 0.001$), two two-way interaction between speaker position and block (X^2 (1) = 116.26, $p < 0.001$) and block and group (X^2 (2) = 39.27, $p < 0.001$). They suggested that signed errors differed between block 3 and block 1 with a greater extent for the pointing (z = 10.99, $p < 0.001$; b1: -0.1°±3.8; b3: -5.7°±12.7) and for naming ($z = 7.64$, $p < 0.001$; b1: -0.6°±5.1; b3: -4.6°±13.3) and with a smaller extent for the reaching group ($z = 2.21$, $p = 0.03$; b1: -0.5°±4.1; b3: -1.7°±6.2). Furthermore, we observed a three-way interaction between block, speaker position and group (X^2 (2) = 23.91, $p < 0.001$) revealing that the effect of speaker position differed between block 1 and block 3 with different extent as a function of group. The lower difference was observed for the reaching group (t = 3.89, $p < 0.001$), while higher differences were observed for pointing ($t = 7.67$, $p < 0.001$) and naming ($t = 10.78$, $p < 0.001$).

2.2. Subjective judgments

By analysing perceived effort and judgments about own performance, we did not document any effect of group (all $ps > 0.26$). We observed higher perceived effort reported during the block 3 (Friedman Test: X^2 (1) = 10.22, $p < 0.001$) and participants reported to performed worse during the block 3 as compared to the first one (Friedman Test: X^2 (1) = 7.91, $p < 0.001$).

2.3. Head-related behaviour

We entered the number of reversals in a GLME model (family poisson) with speaker position, block, side (left, right; encoded as if all participants listened with their right ear in an altered manner) as fixed effects and participant (slope and intercept) as random effects. We found a main effect of block (X^2 (1) = 100.89, $p <$ 0.001) and a three-way interaction between speaker position, block and group (X^2 (2) = 12.76, p = 0.002). The effect of speaker position (slope) differed between block 1 and 3 only for the reaching group (z = 3.56, p < 0.001), but not for the other two groups (all ps > 0.21). Then, we run similar LME analysis on head-rotation extent and found a main effect of speaker position (X^2 (1) = 69.39, p < 0.001), a main effect of block (X^2 (1) = 236.85, p < 0.001), a main effect of side (X^2 (1) = 6.84, p = 0.008), three two-way interaction between speaker position and block (X^2 (1) = 111.50, p < 0.001) and side and block (X^2 (1) = 6.06, p < 0.001) and side and group (X^2 (2) = 7.69, p = 0.02) and block and group (X^2 (2) = 14.35, p = 0.002) and two three-way interaction between speaker position, block and group (X^2 (2) = 24.36, p < 0.001) and side, block and group (X^2 (2) = 9.88, p = 0.007). These results suggested that space explored changed as a function of the side of sounds only for the reaching group when considering block 1 (t = 2.13, p = 0.03, others two *ps* > 0.50) and only for the other two group when considering block 3 (both *ps* < 0.03). We conducted also a similar model on the approaching index, we found a main effect of speaker position (X^2 (1) = 24.72, p < 0.001) and block (X^2 (1) = 136.31, p < 0.001) and side (X^2 (1) = 4.50, p = 0.03), and three two-way interactions between speaker position and block (X^2 (1) = 22.18, p < 0.001), side and group (X^2 (2) = 64.88, p < 0.001) and block and group (X^2 (2) = 54.87, p < 0.001). We also documented a three-way interaction between side, block and group (X^2 (2) = 9.77, p = 0.007). Participants of the pointing group approached more the speaker block 3 as compared to block 1 when they are toward the right as compared to the left (left: t = 9.22, p < 0.001; right:t = 12.36, p < 0.001), while the reaching group approached more the speaker in the block 3 when they were toward the left as compared to right (left: t = 5.08, p < 0.001; right:t = 2.01, p = 0.04).

3. Does sound localization improve across block repetition during simulated asymmetrical hearing loss as a function of instruction (naming, pointing, reaching)? – Head movements

As reported in the manuscript for performance indices, we analysed head-related behaviour by entering into four separate GLME model number of reversal, extent of head rotation, bias of head rotation and approaching index and we considered side of sound (left, right; encoded as if all participants listened with their right ear in an altered manner), group and trial (we combined together block 2 and 3 and considered trials as a continuous variable from 1 to 136) as fixed effects and participant (intercept and slope) as random effect (see Figure S3). Considering number of head reversal, we documented a two-way interaction between trial and side (X^2 (1) = 4.25, p = 0.04) revealing that number of reversals decreased across trial particularly when sounds were emitted by right speakers (altered side). Similar analysis was run on extent of head rotation, we observed a main effect of side (X^2 (1) = 25.92, p < 0.001), two two-way interaction between trial and side (X^2 (1) = 4.53, p = 0.03) and side and group (X^2 (2) = 22.17, p < 0.001) and a three-way interaction between side, trial and group (X^2 (2) = 6.21, p = 0.04). The extent of head-rotation decreased across trials for all groups when sounds were emitted from the left side of the space, while it did not decrease for the naming group when sounds were emitted from the right. The trials' trend differed between left and right only for the naming group (t = 2.13, p = 0.03), but not for other groups (both *ps* > 0.17).

Considering the approaching index, we observed a main effect of side (X^2 (1) = 6.55, p = 0.01), two-way interaction between side and group (X^2 (2) = 83.84, p < 0.001) and a three-way interaction between trial, side and group (X^2 (2) = 10.91, p = 0.004). The trials' trend differed between left and right only for the reaching and pointing group (*ps > 0.05*), but not for the naming (t = 1.15, p = 0.25).

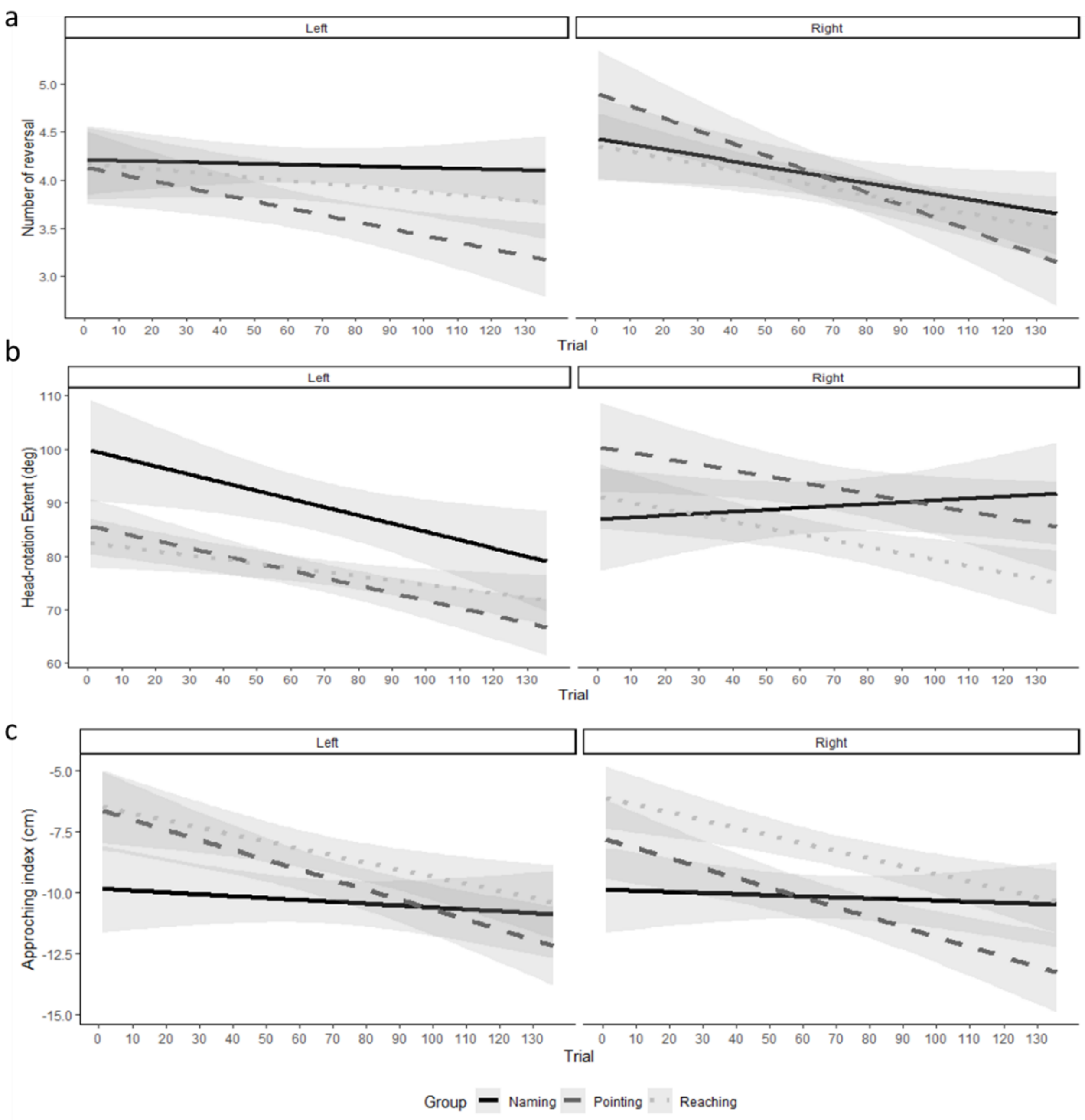

Figure S3. Number of reversals (a), head-rotation extent (b) and approaching index (c) as a function of trials during the altered hearing blocks (x axis), side (left or right) and group (Naming: continue line; Pointing: dashed line, triangles; Reaching: points line). Linear regression (solid line), with 95% confidence intervals.

4. Questionnaire

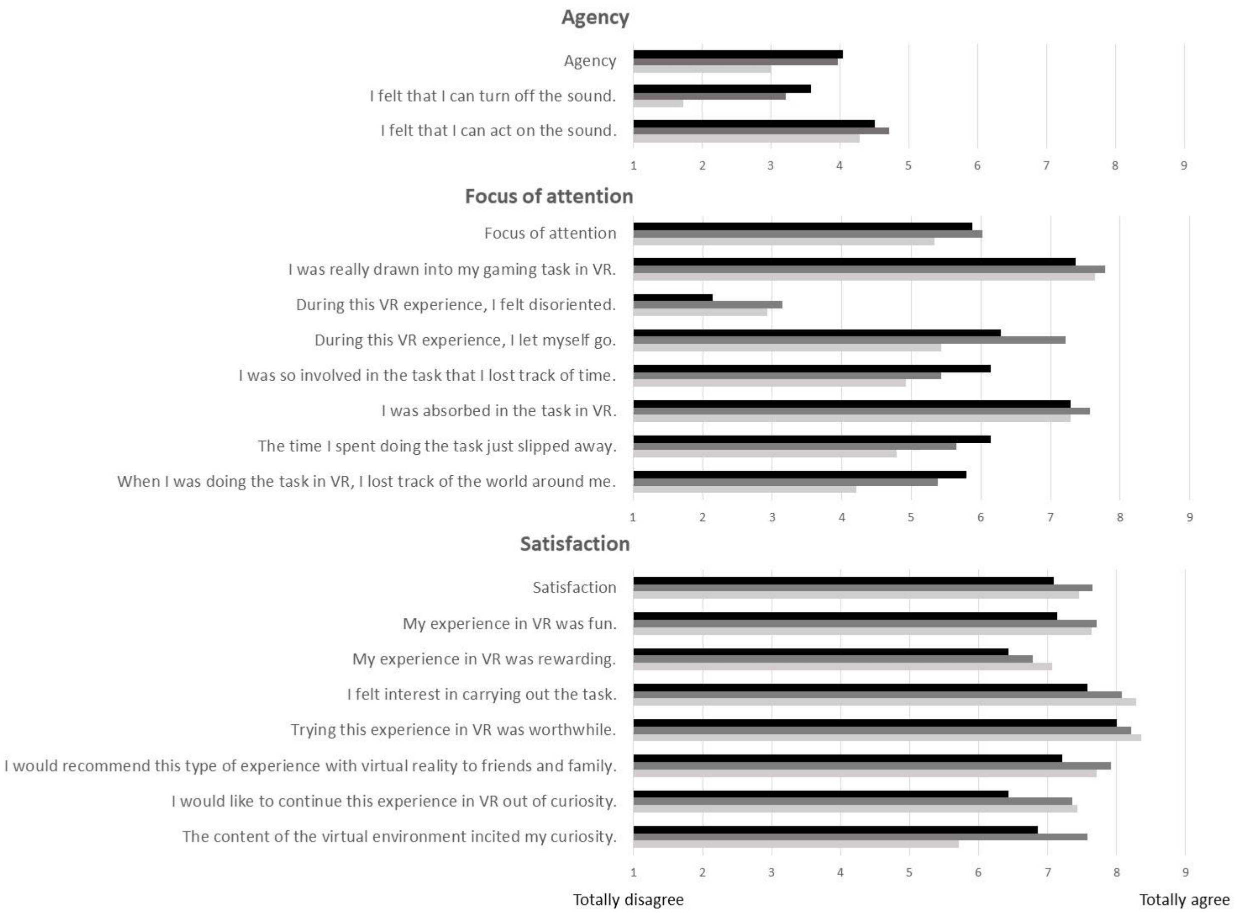

Figure S4. Questionnaire – VR experience. Mean ratings for the Reaching (black bars), Pointing (dark grey bars) and Naming group (light grey bars). Cumulative scores for agency, focus of attention and satisfaction are reported as the first bars of each session separate by titles.
